# Measure of Emotional Episodes with Music (MEEM): Development and psychometric evaluation of a modular instrument

**DOI:** 10.1371/journal.pone.0359288

**Published:** 2026-10-01

**Authors:** Connor Kirts, Suvi Saarikallio, Tuomas Eerola

**Affiliations:** 1 Department of Music, Durham University, Durham, United Kingdom; 2 Department of Music, Arts and Culture, University of Jyväskylä, Jyväskylä, Finland; Universidad Nacional de Tres de Febrero, ARGENTINA

## Abstract

Emotional episodes experienced with music are embedded within the milieu of everyday life, yet most existing psychometric instruments only capture trait-like tendencies rather than state-like contextual factors. In this article, we detail the development of the Measure of Emotional Episodes with Music (MEEM) as a modular solution to capture situational and contextual dimensions of music-related emotional experiences. Building upon prior content-validity evaluation and grounded in the Episode Model, we tested the hypothesised dimensionality for the five constructs across two online vignette experiments. Evaluative confirmatory factor analyses (CFA) in the first experiment supported the assumed latent structure for each of five constructs, showing good model fit and clear differentiation among sub-constructs. Several sub-constructs were refined to more aptly reflect the specific facets captured by the best-performing items. Taking a three item model for each construct forward into a second experiment, CFA again supported the first experiment results and the original hypothesised structure. Associations between the five MEEM scales and two types of emotion ratings (HAAS and GEMIAC) were gathered as an initial indicator of convergent validity. Analysis of a factor structure combining all five constructs did not favour a hierarchical model specified by a single higher-order latent factor but instead a multidimensional interpretation, consistent with Episode Model assumptions. MEEM consists of five separate scales, each representing a theoretically derived construct—with two or three sub-constructs—measured with 35 items in total. Concurrent but distinct development of five constructs permits individualised applications for each of the five scales. This modular design of MEEM allows researchers to administer either the full battery or selected scales depending on their research aims. By targeting emotional states as dynamic, situated, and functionally grounded processes, MEEM provides a meaningful alternative to trait-based instruments, and offers a foundation for future validation and cross-contextual applications.

## Introduction

While nearly every theory proposed to explain music evoked emotion highlights the importance of context [1–3], theoretically driven methodologies capturing functional and goal-based emotional episodes during everyday situations are absent. This is not without substantial effort to operationalise new methods and measurements which classify the emotional experiences people have with music [4]. With endeavours consisting of quick self-report instruments for music-evoked emotions [5], computational prediction of emotional responses to music from audio features [6], and explicating the neural correlates of music-evoked emotions [7]. Researchers, however, often reduce a person’s dynamic, goal-driven, emotional regulation within everyday musical experiences simply because there are no instruments which capture the functional use of music during those situations. A need to understand what function people use music for has also driven several attempts to develop instruments for general motivations [8–11], reward [12], well-being [13–15], mood regulation strategies [16,17], and eudaimonic and hedonic motivations [18]. Unfortunately, these instruments have been developed to only classify general listener traits and not intended to capture the functional use of music during situated states. Without a state-based instrument, describing and understanding the purpose for a person’s emotional episode is not possible.

Traditionally, emotions are conceived as classical categories where each instance is defined by a shared underlying mechanism and diagnosed by a stable or coordinated set of observable features (reported feeling, facial, physiological, neural) and variation is considered noise, even across different contexts [19]. Research on music and emotion have adopted what Barrett calls “emotions as natural kinds” [20], where specific combinations of triggers and mechanisms are assumed to be responsible for certain emotions [21,22], evident by a number of empirical studies over the years attempting to uncover the feature criteria to diagnose specific emotions (e.g.[4,5,23]). This perspective, however, appears outdated as it is no longer supported by evidence from neuroscience [24–27] and behavioural studies [28,29]. A key missing perspective is that instances of emotion are situated—constructed and categorised ad hoc by the person—based on the function or goal for the person at the time [30]. An implication of this constructionist paradigm shift (see [29]) has reconceptualised music-related emotional experiences as being embedded in and constructed by psychological and social needs that vary from situation to situation [31–34]. This theoretical change calls for rethinking methodology and devising new instruments which capture functional and situated uses of music to explain why emotional episodes are meaningful for a person in a particular situation.

The Episode Model [34] is an example of such a theory, requiring the development of new methodologies, in music-emotion research which conceptualises emotional episodes based on the function of music as it pertains to reflecting a listener’s goals and engagement in a given moment. The model distinguishes between five prototypical episode functions commonly attributed during music listening: Enjoyment–Distraction–Relaxation (EDR), Connection–Belonging (CB), Focus–Motivation (FM), Personal–Emotional–Processing (PEP), and Aesthetic–Interest–Awe (AIA). Taken together, the five episodes provide an account of music-related emotions through the functional features people report within situated experiences [35]. Rather than isolated affective responses defined by classical emotion categories, there is now the need to explain how a person conceptualises their situated experience as it pertains to their self-regulation of emotion with the music. In an effort to account for how a person understands the music within the situation, configurations of emotional tone (e.g., qualia, core affect), induction mechanisms (e.g., neurological, behavioural, and physiological changes), listening modes (e.g., attention, agency), and contextual meanings (e.g., appreciation, familiarity) are descriptive schemes which further differentiate between and within the prototypical episode functions [34]. Explaining the situated functional features of an instance of emotion is hypothesised to depict the procedural nature of emotions during music listening as dynamic, reflecting a person’s ongoing self-regulation, for hedonic regulation, social affiliation, task-related focus, self-reflection, and aesthetic exploration.

Music-related emotional episodes, as expressed in the Episode Model [34], are only theoretical descriptions of the common categories of functions associated with emotional experience during music listening. Work has been done to formulate 25 latent constructs that are reflective of the five episodes of the Episode Model; these constructs have been operationalised into items and evaluated by 14 subject matter experts across two rounds of evaluation [36]. Kirts and colleagues [36] operationalised the relevant constructs from the Episode Model, defining what content is assumed to represent these constructs. Following this, the authors generated content to broadly reflect these constructs and had experts evaluate whether this content is suitable through both quantitative ratings of each item and qualitative data on the overall relevance of the content for each sub-construct (see [36]). The 14 experts were published authors on topic areas related to the constructs, primarily from Europe but several were from North and South America, Asia, and Australia. These experts evaluation process followed the CVI recommendation [37], where the expert assessments of relevancy of the proposed items were rated on a scale of 1–4 (1 = not relevant to 4 = extremely relevant), where the items taken forward received item-level indications of content relevance (I-CVI) above .70 [38]. This process determined 23 constructs that were satisfactory (scale level values of content relevance, S-CVI, were .93 across all constructs, indicating excellent content validity, see [37]) at retaining the theoretical position of the Episode Model and its five prototypical episodes while sculpting the content for clarity [36]. This critical piece of evidence provided suitable candidate items that are assumed to reflect their constructs.

With only these expert opinions, however, we do not know whether the best identified items deliver adequately the proposed structure of the five constructs suggested by the Episode Model. As best practice guidelines for psychometric development reiterate, multiple pieces of evidence—alongside theory—are needed to support the interpretations of measurements for proposed purposes [39–44]. As development is an iterative process, we are moving on from expert assessment to investigate whether laypeople respond to the items and testing whether the proposed dimensionality of the constructs can be replicated and the appropriate items. Actual responses to the items in a variety of contexts are needed to determine whether the items – and which subset of items – represent the proposed structure. Questions concerning the frequency of episodes in everyday life, how episodes emerge from the perception of situational variables, what correlates differentiate participant experiences further, and how the episodes can be used for predictive purposes, cannot be investigated without establishing an instrument with scales representing each of the constructs. However, we seek to establish initial evidence for the validity and reliability of the instrument being developed alongside testing the dimensionality, in line with best practice recommendations [39–41,44]. Continuing from the operationalisation done by Kirts and colleagues [36], producing separate self-report scales for each episode is a critical step to enable further evaluation and implementation of the Episode Model in future research. As the model was operationalised with a modular design and hierarchical constructs, MEEM—Measure of Emotional Episodes with Music—should reflect this theorised structure.

The aim of the current work is best understood through the overarching aim to develop a self-report instrument to capture the presence of emotional episodes which encompass the functional construction of individuals’ emotional experiences with music in everyday life. Here, we continue on from the establishment of suitable content assumed to reflect each of the components of the individual episode constructs: Enjoyment–Distraction–Relaxation (EDR), Connection–Belonging (CB), Personal–Emotional–Processing (PEP), Focus–Motivation (FM), and Aesthetic–Interest–Awe (AIA). We aim to develop five separate psychometric scales, as the episodes reflect separate constructs, with content (a total of 87 items) differentiated through 2–3 sub-constructs defined in a prior study [36]. The current study aims to finalise the content for each episode construct by determining the more discriminating items, reliability, and psychometric robustness for each scale and its components. Here, we evaluate the content of the episode constructs and sub-constructs in two experiments. In Experiment 1, all candidate items for each construct will be examined in a rating experiment that elicits specific situations and functions using vignettes. This stage will test whether the latent factor structure of each episode type reflects its theoretical conceptualisation, identify the items that best represent the proposed sub-constructs, and evaluate their internal consistency and reliability. In Experiment 2, the optimal items from all episode types will be administered jointly in a rating experiment using contextualised vignette-based scenarios. This will allow the factor structures identified in Experiment 1 to be reassessed and potentially supported, as well as enable evaluation of discriminant validity among sub-scales and estimation of convergent validity with established emotion measures.

## 1 Experiment 1

This experiment was designed to investigate whether the proposed latent structure can be obtained with a reduced set of items while maintaining theoretical, statistical, and pragmatic considerations [45]. As there were five major constructs to develop, we conducted this process five times to explore the dimensionality for each construct separately. To assess which items should be retained for each construct, we relied on methods of internal consistency (e.g., factor analysis). We also carried out reliability evaluations for responses to the sub-constructs and tested the measurement invariance of the obtained structures.

Considering the content and proposed structure for each of the five constructs was evaluated by content experts prior [36], we had reasonable justification to test whether this assumed structure would be replicated with real responses [46]. Proposed factors, therefore, were constrained as is typical for confirmatory factor analysis (CFA), to reflect the theoretical structure of each construct specified during operationalisation (i.e., three factors for EDR and AIA, two for FM, CB, and PEP). For reference, Fig 1 provides an overview of the proposed structure for the five constructs with sub-constructs acting as the latent factors. Cross loading was allowed within the factor constraints to investigate the extent to which any strongly correlated items affected the structure between sub-constructs within each of the five constructs. As the items were developed through a systematic expert content validation process, established evidence supports our a priori hypotheses about which items should load onto each sub-construct and the factor structure of the constructs [36]. An evaluative CFA was therefore an appropriate method for testing whether these theoretically specified relationships held in the empirical data [46–48]. Moreover with this evaluative CFA we would be able to identify weak items and assess the optimal model fit. We followed recommendations for item retention, including keeping items with communalities above .60 and keeping items which load above .40 that do not cross loading above .30 [49]. It is important to acknowledge, however, such a process is exploratory and so we will take whatever the optimal item configuration is obtained in this experiment and retest it, with a new sample of participants, as is standard practice [44,46]. In our case, the expert validation process had already established the theoretical structure and a clear ranking of items for each latent factor [36], making a true EFA redundant and potentially misleading for each of the five constructs [50].

**Fig 1:**
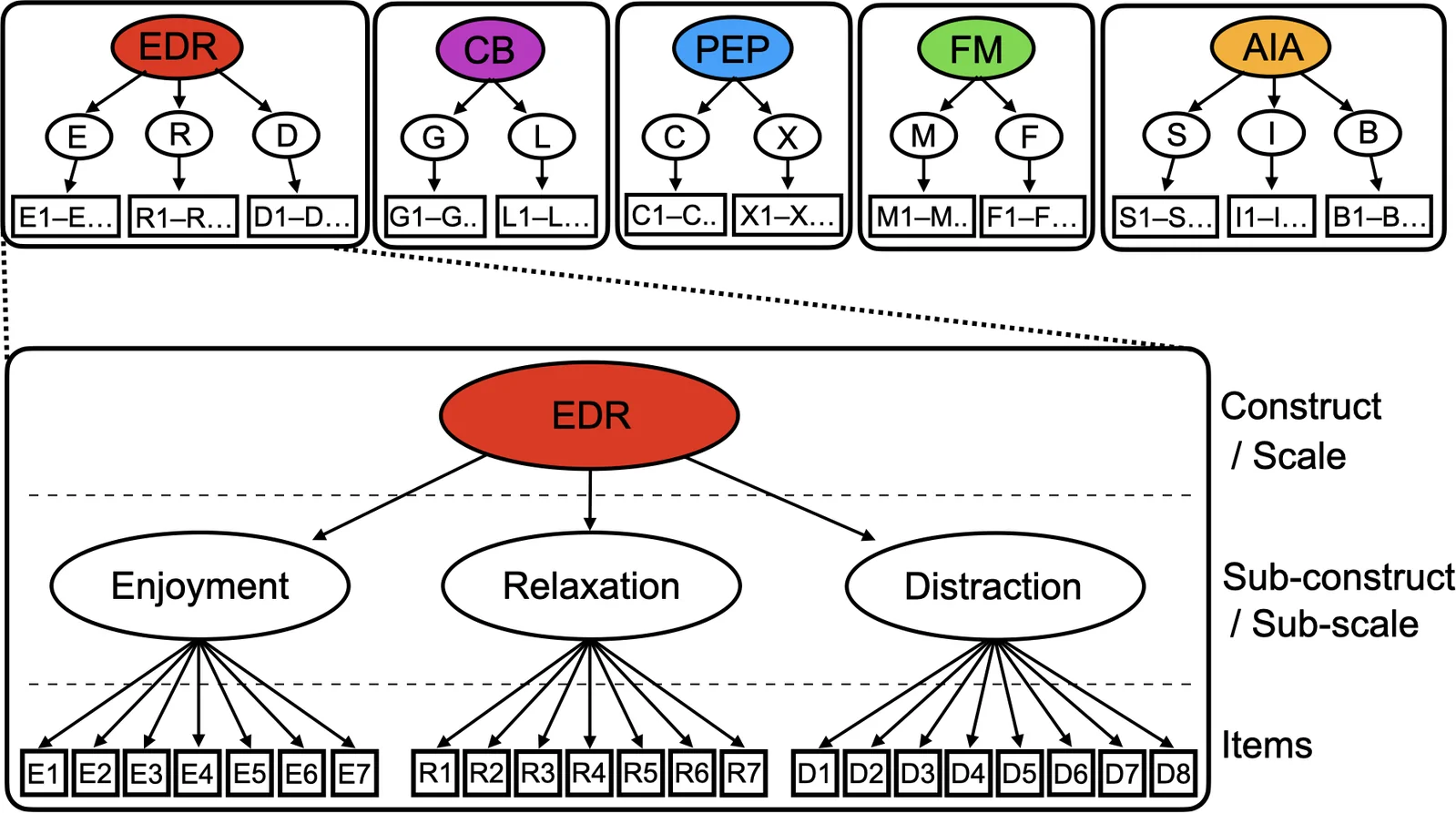
Overview of the theoretical structure for the five episode constructs. The constructs are made of either two or three subconstructs. The example provided showcases the Enjoyment-Distraction-Relaxation (EDR) construct with its assumed structure following from the content validity process. Sub-construct IDs are provided in lieu of their actual names for brevity for the other construct diagrams.

As the purpose of the instruments is to capture emotional experiences related to music in different situations and contexts, it is important to provide appropriate context for rating the items. For this reason, we applied a vignette method instead of relying on decontextualised ratings [51]. Vignettes will provide concrete contextualised scenarios that approximate real-world situations. This approach embeds the items within realistic situations, allowing participants to demonstrate how they would actually respond more so than how they think they might respond in the abstract without context [52]. Asking participants to evaluate their experience within scenarios reduces social desirability bias—a particular concern when measuring attitudes and emotions [53]. For the purpose of developing scales to represent the five constructs, we are using this vignette method to broadly establish suitable measures while controlling the situational variables. Our constructs about emotional experiences are inherently situational, requiring concrete contexts to meaningfully assess their differences and similarities. The vignette method allows us to capture these context-dependent responses while maintaining the rigor of confirmatory factor analysis but future validity and reliability work will have to test these constructs in real world scenarios (for vignettes, see S1. Appendix 1).

### 1.1 Method

#### 1.1.1 Ethics declaration.

This study was approved by the Music Department ethics committee at Durham University (MUS-2024-01-24T18_29_22-bmgm21). Experiments were conducted between 11/08/2025–03/09/2025 in accordance with the ethical standards of the Declaration of Helsinki for research involving human participants. All participants provided voluntary written informed consent and had the option to withdraw from the study.

#### 1.1.2 Procedure.

To evaluate the dimensionality for each of the five constructs, we investigated the assumed structure of each construct in five sub-experiments. We conducted these experiments online, using the survey hosting platform Qualtrics and sampling voluntary participants from Prolific. Participants who decided to take part were asked to provide consent after reading the experiment’s full information sheet. Demographic questions concerning age, gender, and musical sophistication, were obtained after participants provided informed consent. We sought to develop five scales simultaneously with the same procedure, methods, and analysis.

One major concern for the initial scale development process is sample size. Commonly cited guidelines suggest minimum samples of 100–150 for CFA [54]. Some researchers argue that samples as small as 100 are adequate when item communalities are high [55]. Here, we evaluated communalities to determine whether our initial sample size of 100 participants per sub-experiment was adequate. Another aspect to consider is the participant to parameter ratio [56]. Considering our models ranged from 19 to 27 estimated parameters, we exceed the recommended minimum ratio of 5:1 with a sample size of 100 [56]. In each sub-experiment, we asked the participants to rate items against vignettes—one vignette for each sub-construct that captures transient state rather than a stable trait theoretically designed to elicit the target state —so we obtained multi-level data from each participant. The repeated observations were therefore not intended to increase the effective sample size but to sample the latent construct across multiple situational contexts, reducing vignette-specific variance and allowing the CFA to estimate the construct independently of any single eliciting scenario. Such multi-level data can be modelled with a CFA technique using robust standard errors that adjust for the nested data at the participant level.

For the task, participants rated the item sample for the episode construct (22 items in the case of EDR, see Supporting Material) after reading a vignette (see S2 Appendix 2). The presentation order for vignettes was randomised in each of the five surveys. Each participant was presented with all corresponding vignettes for that construct in a given survey. A participant doing the EDR survey, for example, would see three vignettes and answer the 22 EDR items after each vignette. Attention check questions were intermixed within the randomised order of items for each survey. After answering all of the items for a given vignette, participants would indicate how relevant they considered the vignette to be based on their own life experience. After completing all the items for each vignette in a given survey, the participants were routed back to prolific. Participants were not allowed to complete another sub-experiment if they had already participated in one of the five surveys.

#### 1.1.3 Modelling.

To determine the goodness of fit of the factor structure of these previously evaluated and ranked items representing sub-constructs (2–3) for each of the five constructs, we applied multi-level confirmatory factor analysis (CFA) using the lavaan package in R [57]. For each sub-experiment (one sub-construct), observations were nested within individuals to vignettes (2 or 3 vignettes, one for each sub-construct), and the multilevel structure was modelled by specifying participants as clusters. This multi-level modelling approach allows us to evaluate whether the total variance in the observed items is due to variance in responses to the vignettes (within-person variance) versus how much the structure varies between different people. Two (FM, CB, PEP) to three (EDR, AIA) latent factors were specified, each measured initially by all available observed items (S2 Appendix 2) or by exploring a model using the three items with the highest factor loadings for each sub-construct. The purpose of the three items per latent factor was to explore a more parsimonious model following the practical guidance [58,59]. This vignette design is consistent with our theoretical premise that affectual responses during emotional episodes are context-dependent [34,36], though the present manuscript is not intended to isolate any specific situational features affecting within-person variability. Rather than treating this variability as pure measurement error, we retain within-person variance in the multi-level model and report relevant reliability metrics but do not interpret it as a within-person model. All five models were estimated using the robust maximum likelihood estimator (MLR), with participant ID specified as a cluster variable to adjust for non-independence. Considering participants responded to multiple vignettes in a random order with the same construct relevant item content, the use of MLR corrected for our nested/clustered multi-level approach, potential standard errors, and the $\chi^{2}$ non-normality statistic. The particular vignettes, however, are not assumed to be naturally occurring higher-order factors, but rather allow for repeated measurement occasions. Model fit was evaluated using a standard combination of absolute and comparative fit indices ($\chi^{2}$, CFI, TLI, RMSEA, and SRMR). All data and analysis notebooks are available at https://zenodo.org/records/21909314. This multi-level modelling approach aligns with our underlying theory which specifies that emotional episodes, the affectual response and the contextualisation of this response, will vary depending on what situation a listener finds themselves in [34,36]. While the constructs are meant to operate in this manner, it is not known how the situational variance would manifest in a given situation for a particular person.

#### 1.1.4 Materials.

The sub-constructs each had one vignette (12 total) which were designed to be typical situations that would garner responses to items associated with a specific sub-construct (see S1 Appendix 1). The episode construct EDR, for example, had three vignettes corresponding to three sub-constructs: “You are attending a celebration where music is the heart of the party. You are able to join in the fun which is aided by the music throughout the event” for Enjoyment, “It was pointed out that you missed an important detail in your work which you now have to redo. You put on a music playlist to drown out self-criticism as you fix the mistake” for Distraction, and “After a long and demanding week, you take time to rest by listening to music. The stress melts away and you start to recover as you listen along” for Relaxation (see S1 Appendix 1 for all the vignettes).

#### 1.1.5 Measures.

Each episode construct was probed with appropriate items obtained from expert content evaluations ([36]; 22 for EDR, 14 for FM 16 for CB, 20 for PEP, 15 for AIA). Each item is worded to reflect current or recent experiences (e.g., The music made me feel calm or relaxed; see S2 Appendix 2) and it is expected that the instrument would be administered to people during or immediately following experiences with music. Considering assumed response behaviour, participants indicate whether they are experiencing the measured attribute described by each item on a five point scale from Strongly disagree (1) to Strongly agree (5), following best practice recommendations [40,60].

One content domain, the AIA’s Curiosity sub-construct had less than three items following the expert content evaluations. Typically, if there are less than three items for a supposed factor then there are potential issues that relate to factor complexity and no zero elements in the coefficients ([61], p. 244). These were, however, not issues with the factor case. Nevertheless, to circumvent potential issues of only having two items as indicators of a sub-construct before conducting the CFA, we included borderline unacceptable items from the expert content evaluations to bolster the number of items in this sub-construct.

To review if the participants found the vignette believable, we obtained ratings of relevance on a four point scale (1 = not relevant to 4 = extremely relevant). This was used to gauge whether the vignette was something participants believed as a hypothetical experience they could imagine themselves experiencing.

As a demographic variable, we collected an indication of participants’ musical interest and experience through one item from the Ollen Musical Sophistication Index (OMSI; [62]). Common use of this single criterion indicator is the result of work of investigating two popular music sophistication indexes to determine a single best estimating item to indicate overall sophistication [63]. The resulting OMSI item, “In terms of your musical interest and expertise, which title best describes you?”, was best and is widely used to demarcate between musician and non-musician groups or between those who are assumed to have little, moderate, or high levels of interest in music. There are six self-selected options, “non-musician”, “music loving non-musician”, “amateur musician”, “serious amateur musician”, “semi-professional musician”, and “professional musician”. In this article, we use the OMSI variable to indicate between musician and non-musician groups.

#### 1.1.6 Participants.

Our sampling excluded those outside of the United Kingdom, if they had already participated in another episode survey, and if they had poor quality Prolific ratings. There were 587 participants sampled, of which, 4% were filtered out due to incompletion, 5.6% failed the attention check questions, and another 4.9% were removed following correlation to the group mean (threshold of 0.001), this left N = 501, (N = 100 for each episode construct, except AIA which had N = 101; $M_{age}$= 42.6, ${SD}_{age}$= 12.9, 54.5% females, 18% musicians). The five sub-experiments did not differ statistically in terms of age or musicianship (Age: F(4,495), *p* = 0.19; OMSI: $\chi^{2}$(4)=24.8, *p* = 0.21) although one sub-experiment (#4) significantly deviated in gender distribution from 50/50 (69/31, $\chi^{2}$(1)=6.73, *p* < 0.01, while the rest of the sub-experiments had nearly equal distribution (41%, 55%, 50%, and 58%).

### 1.2 Results

Relevance ratings for the vignettes of each episode construct showed that participants considered all vignettes believable as potential experiences (three EDR vignettes M = 2.92, SD = 0.86; two FM vignettes M = 2.99, SD = 0.94; two CB vignettes M = 2.72, SD = 0.99; two PEP vignettes M = 2.79, SD = 0.91; three AIA vignettes M = 2.58, SD = 0.89). Looking into the reliability of the ratings of the items within each construct and vignette was calculated using multi-level reliability from the psych library (version 2.5.3; [64]), where the vignettes provided the within-subject unit. Using the $R_{kF}$ metric that captures the reliability of average rating across items and times, all constructs scored above 0.95 [65]. In terms of between person differences across vignettes ($R_{kRn}$ in [65]), the reliability scores range from 0.52 for FM to 0.87 for AIA. Both metrics suggest moderate to excellent multi-level reliability for the five constructs generalised at the between participant level. Looking at reliability metrics at the within-person level, when variation is averaged over items so that responses to vignettes are nested ($R_{cn}$ [65]) the reliability ranges from 0.76 for AIA to 0.93 for EDR.

#### 1.2.1 CFA descriptives.

There were 87 items in total assessed across the five CFA processes (see S2 Appendix 2 for detailed descriptives). The outcome of the CFA for the EDR sub-experiment, all three sub-constructs appear to have at least three suitable item candidates with factor scores ranging from .54–.79 for Relaxation, .73–.84 for Enjoyment, .64–.77 for Distraction. The Connection–Belonging two factor structure between the Group Cohesion / Socialisation and Reducing Loneliness sub-constructs saw candidate items for the former ranging from .61–.85 while candidates ranged from .84–.89 for the latter. The two factor structure for the Focus–Motivation construct was supported but items diverged slightly from the prior expert classification [36]. The Energy Control sub-construct had two strongly loaded items from the Focus sub-construct which both mention motivation; (F1) “I used the music to motivate myself”, and (F7) “The music motivated me to finish the task I had to do” (see S2 Appendix 2). With this in mind, the top three candidate items for Energy Control ranged from .84–.87 (M1, M3, & F1/M9) and suitable candidate items ranged between .70–.87 for Focus. Strong loading candidate items for the Personal Emotional Processing construct ranged .62–.82 for Reflection / Coping sub-construct and .64–.85 for the Expressing Feelings sub-construct. Finally, the Aesthetic–Interest–Awe three factor structure had suitable items with strong factor loadings for Being Moved / Spirituality ranging .61–.89 and .62–.82 for the Aesthetics sub-construct. The Curiosity sub-construct was identified coincidentally by a factor with the two items which were originally identified by the experts as best. These items had strong loadings (.83 for I2 and .85 for I1, see S2 Appendix 2) and, despite adding additional items, were again the top two indicators for the Curiosity sub-construct.

#### 1.2.2 CFA model fits.

As expected, the full suite of items (22 for EDR, 14 for FM, 16 for CB, 20 for PEP, 15 for AIA) yielded inadequate fits across most constructs (**EDR** TLI = 0.926, $\chi^{2}$=581.9, *p* < .001, CFI = 0.934, RMSEA = 0.078, SRMR = 0.071, **FM** TLI = 0.893, $\chi^{2}$=291.5, *p* < .001, CFI = 0.912, RMSEA = 0.133, SRMR = 0.076, **CB** TLI = 0.741, $\chi^{2}$=676.9, *p* < .001, CFI = 0.778, RMSEA = 0.167, SRMR = 0.117, **PEP** TLI = 0.852, $\chi^{2}$=578.6, *p* < .001, CFI = 0.868, RMSEA = 0.110, SRMR = 0.066, and **AIA** TLI = 0.810, $\chi^{2}$=702.0, *p* < .001, CFI = 0.840, RMSEA = 0.140, SRMR = 0.087). None of the constructs obtain adequate measures of fit, except for satisfactory RMSEA and SRMR for EDR. The full models are overspecified, too complex or the items simply are not tapping into the latent factors as expected from the expert evaluations.

When a similar analysis is carried out using the three (or two in the case of the Curiosity sub-construct) items scoring highest in factor loadings, good model fits are obtained across the board (**EDR** TLI = 0.975, $\chi^{2}$=51.85, *p* < .001, CFI = 0.984, RMSEA = 0.062, SRMR = 0.038, **FM** TLI = 0.985, $\chi^{2}$=16.58, *p* < .035, CFI = 0.992, RMSEA = 0.073, SRMR = 0.018, **CB** TLI = 0.986, $\chi^{2}$=13.85, *p* < .086, CFI = 0.992, RMSEA = 0.060, SRMR = 0.041, **PEP** TLI = 0.979, $\chi^{2}$=15.92, p < .044, CFI = 0.989, RMSEA = 0.070, SRMR = 0.027, and **AIA** TLI = 0.966, $\chi^{2}$=54.76, p < .000, CFI = 0.979, RMSEA = 0.086, SRMR = 0.051). The full breakdown of the items, sub-constructs and metrics for each construct is available at https://zenodo.org/records/21909314. All models demonstrated acceptable to excellent fit. CFI values ranged from .979 to .992 and TLI values ranged from .966 to .986, exceeding the recommended threshold of .95 [66]. RMSEA values ranged from .060 to .086, with four of five models meeting the stringent criterion of .06 and all falling within the acceptable range of .08 [67]. SRMR values ranged from .018 to .051, all well below the recommended cutoff of .08 [66]. The chi-square tests were significant for two models (EDR and AIA), which is common with adequate sample sizes and is not considered problematic when other indices indicate good fit [68].

#### 1.2.3 Measurement invariance.

To explore measurement invariance across vignettes, we compared models with increasingly restricted models (configural, metric, and scalar invariance) for each construct [69]. With our vignette design, we expect that the configural invariance for each construct will be achieved to demonstrate that the items represent the latent constructs similarly. We also anticipate that the measurement invariance holds for metric invariance, as it would suggest comparable relationships with the latent variables across the vignettes. However, the more restrictive scalar invariance is likely to be unachievable as the vignettes themselves produce variation in the intercepts even if the structure is otherwise similar. We analysed measurement invariance with a robust $\chi^{2}$ comparison between the original and each restricted model (see [70]). All constructs pass the *configural invariance* test, suggesting that the overall structure is valid across the vignettes when coefficients are allowed to freely vary (EDR *p* = .942, FM *p* = .612, CB *p* = .995, PEP *p* = .977, AIA *p* = .121, each *p* value comparing the full model with configural model with robust $\chi^{2}$ comparison). Moving on to *metric invariance* tests, where loadings are fixed to be the same across the vignettes, 3 out of 5 constructs pass this comparison (FM *p* = .897, CB *p* = .156, and AIA *p* = .614). However, none of the constructs passed *scalar invariance* tests, where not only loadings are constrained to be equal across the vignettes but the intercepts are also fixed to be equal across vignettes. The scalar invariance could be attempted to be recovered by allowing some items to be released from the constraints in the model, but our theorising about the vignettes suggested that the item loadings can vary across the vignettes despite them maintaining the overall structure across the construct, so neither scalar nor metric invariance was a desirable quality in our design.

#### 1.2.4 Sub-construct interpretation.

While our empirical process has indicated suitable candidate items for each of the constructs, the reduction of content has influenced the existing conceptualisation for some of the sub-constructs from the prior assumptions [36]. Our choice of approach, both in terms of methodological approach and retention of the 3 best items, has emphasised narrower aspects of the sub-constructs. With this consideration, it is important to reflect on whether the items still target the intended content domains stipulated by Kirts and colleagues [36].

Energy Control, as a sub-construct of Focus–Motivation, was originally operationally defined as “Music used to control levels of intensity or drive, pumping up, motivation, stimulation” [36], but the resulting best items primarily referred to a narrower concept akin to “motivation”. Hence we have relabelled the sub-construct hereafter as Motivation to address this. A similar result occurred with two facets of Aesthetics–Interest–Awe; “Being Moved / Spirituality” and “Aesthetics” sub-constructs. Originally, “Being Moved / Spirituality” referred to “Music used to connect to a higher ideal or abstract feeling of non-social connection, religious activity, feeling or seeking strong emotions” ([36]) but the resulting items only contain spirituality related items, hence we relabel the sub-construct as Spirituality. Similarly, Aesthetics referred to “Music used to achieve or create a desired ambience, fit to the ‘mood’” but the best items refer primarily to a narrower concept of beauty. We therefore labelled the sub-construct as Beauty. The last relabelling is for the Reflection / Coping sub-construct of Personal–Emotional–Processing which originally refers to when “Music [is] used to remember or to associate to other objects, provide a sense of comfort, processing experiences”. The resulting best items from the empirical evaluations refer to comfort rather than to coping or reflection, so we renamed the sub-construct Comforting. Finally, for consistency, we simplified an existing label from Group cohesion/socialisation to Group bonding to avoid confusing labels. The purpose for relabelling of the sub-constructs is to acknowledge that the empirical data and the analysis procedure has influenced, mainly trimmed and focussed, the emerging latent constructs. To retain clarity, we have updated the labels of these specific sub-constructs to reflect the more narrow aspects of the content which emerged following Experiment 1.

### 1.3 Discussion

The consistency in participant ratings and the structures imposed suggest that most of the constructs can be adequately captured by a small number of items from each sub-construct. In essence, this supports the theoretical structure put forward in the theory [34] as operationalised through representative content evaluated by experts [36]. Each construct is now adequately empirically defined and the best items provide a good to excellent account of the latent factors. FM, CB, and PEP show the best fit overall, and while AIA is at the upper boundary of what is acceptable fit, the majority of the fit measures provided strong support for AIA as well. The decision of using the three items receiving highest loadings is somewhat arbitrary and can be challenged for another exploration using the best four or even five items. Nevertheless, this is not an uncommon operation in scale development literature to reduce the pool so that inadequate and cross-loaded items are removed ([40], p. 11), which is desirable also from the practical point of view. Given that our items were developed through a rigorous expert content validation process, we had strong a priori theoretical expectations about the factor structure which was supported by our findings here. Confirmatory factor analysis was therefore more appropriate than exploratory factor analysis, as it allowed us to test whether the empirically observed relationships among items matched the theoretically specified structure [47, 68]. After the analysis of the results and observing the contents of the best items representing the constructs and sub-constructs, we decided to relabel the sub-constructs to better reflect the items remaining in the factor. This relabeling should aid in future interpretations of the instrument and remind that there can be subtle “construct drift” [39] or narrowing down of the construct during the item reduction [58].

Our sample size of 100 participants, while modest, is adequate for the confirmatory factor analyses we conducted. Simulation studies demonstrate that sample size requirements for CFA depend on model and data characteristics rather than fixed thresholds, with samples of 100 producing reliable results when factor loadings are strong and models have simple structures with multiple indicators per factor [71,72]. Our models met these conditions, with strong factor loadings (range: .65–.95). The participant-to-parameter ratios (ranging from 10:1–16:1) exceeded recommended minimums [56], and the consistent model fit across all five constructs provides empirical evidence that our sample was sufficient for these analyses. Nonetheless, we acknowledge that replication with larger samples would strengthen confidence in these five measurement models. It would be especially beneficial to 1) gather responses across a variety of real world situations, 2) evaluate responses as within participant data, and 3) determine whether different populations understand the item content differently. Before these future steps, however, it is important to investigate whether the structure replicates with an independent sample.

## 2 Experiment 2

In order to corroborate and further validate the structures of the five episode constructs, we took the three best-performing items identified in Experiment 1 and subjected these to a new vignette experiment with a new sample of participants. It is important to reassess dimensionality of the five structures that we found corroborated the theoretical and content validity stages considering Experiment 1 explored retaining different items in the structure [44, 46].

### 2.1 Method

In this new experiment, we used a multi-group CFA technique to investigate the dimensionality for each sub-construct. Instead of having participants respond only to the items assumed to reflect the sub-constructs, this experiment included all 35 items. We designed this experiment to tackle each of the sub-constructs separately, with 100 participants per sub-experiment (12 in total). The same vignettes used in the first experiment were used again to be consistent. The procedure, measures, and materials remained largely the same with the exception of removing the quality of vignette questions and incorporating measures to evaluate convergent validity between the sub-constructs and affectual states.

In an effort to establish preliminary convergent validity evidence for affectual states, we used two state-based instruments: core affect and a music specific emotion labeling scheme. First, the Hedonic and Arousal Affect Scale (HAAS) is an instrument that measures bidimensional core affect (i.e., valence and arousal) with 12 emotion adjectives as items [73]. Items are rated on a 5 point scale (0 = not at all, 1 = slightly, 2 = moderately, 3 = very, and 4 = extremely) to reflect the extent adjectives describe how participants felt. Second, the GEneva Music-Induced Affect Checklist (GEMIAC) is a checklist of 14 adjectives that are commonly reported when people describe their induced emotion from music [74]. Participants select the items which describe their experience with music and include compound concepts like “Filled with wonder, amazed” and “Melancholic, sad”. For the GEMIAC checklist, we also included an option where participants could select “None of these”. Half of the sub-experiments (6) used HAAS, and half (6) GEMIAC, and these ratings were given after rating the items to avoid any influence of the emotions spilling into the assessing the statements.

#### 2.1.1 Participants.

1287 participants were recruited from Prolific in total, each completed one of the 12 sub-experiments. One participant did not complete, 44 were inattentive, across the 12 vignettes 39 were removed for reliability, and 3 last participants were eliminated for vignette 2 because of over-recruitment. Overall, 1200 participants (N = 100 for each sub-construct) completed the experiment. Age M = 44.69, SD = 13.39, 50.5% females (11 non-binary/prefer not to say), 19.25% musicians (amateur to professional from OMSI), 3.7% failed attention check (before reliability trimming). Demographic subsets for constructs do not differ statistically (gender *p* = 0.25, age *p* = 0.87) except for musical expertise ($\chi^{2}$=21.76, *p* < .05), where one of the episodes (sub-construct Expression) had 28% musicians compared to the overall (19.25%). HAAS ratings were given by a total of 639 participants and the GEMIAC checklist was used by 561 participants.

### 2.2 Results

We applied multi-group confirmatory factor analysis with vignettes as the grouping variable (12 groups, N = 100 each). This approach accounts for systematic differences in response patterns across experimental scenarios while allowing us to test measurement invariance of the factor structure. We applied CFA with lavaan using robust maximum likelihood estimator (MLR), allowing the covariance between the factors to be unspecified [57]. We report each construct separately as separate scales. Data and analyses are available at https://zenodo.org/records/21909314.

#### 2.2.1 Scale for Enjoyment, Distraction and Relaxation (EDR).

The three latent factors corresponded to the supposed sub-constructs representing EDR, Enjoyment (E), Distraction (D), and Relaxation (R), and were captured with three items for each latent factor. The standardised coefficients are shown in Fig 2. The CFA model was excellent, all measures of fit provided evidence that the latent factors and the items capture the variance of the statements well ($\chi^{2}$=89.9, *df* = 72, *p* = .074, TLI = 0.976, CFI = 0.984, RMSEA = 0.050, SRMR = 0.046). Items related to enjoyment such as E1, E6 and E7 loaded strongly on the same factor, as expected. The items predicted to be strong for the Distraction sub-scale, such as D1, D6 and D7, received positive loadings to the same latent factor. Also the Relaxation sub-scale obtained consistent positive loadings from the relaxation items (R1, R3, and R7). Tests of measurement invariance compared the model across the different groups, here (and for all of the other scales) we use the vignettes as our grouping. With an equal factor structure across the groups (i.e., configural invariance), the EDR scale passes this test based on the model fit. Adding stipulations that the factor loadings should be equal across groups (metric invariance) and that both loadings and intercepts should be equal (scalar invariance) were also tested. The EDR scale passes the metric invariance test using the robust $\chi^{2}$ (*p* = .43) but fails the scalar invariance test (*p* < .001).

**Fig 2:**
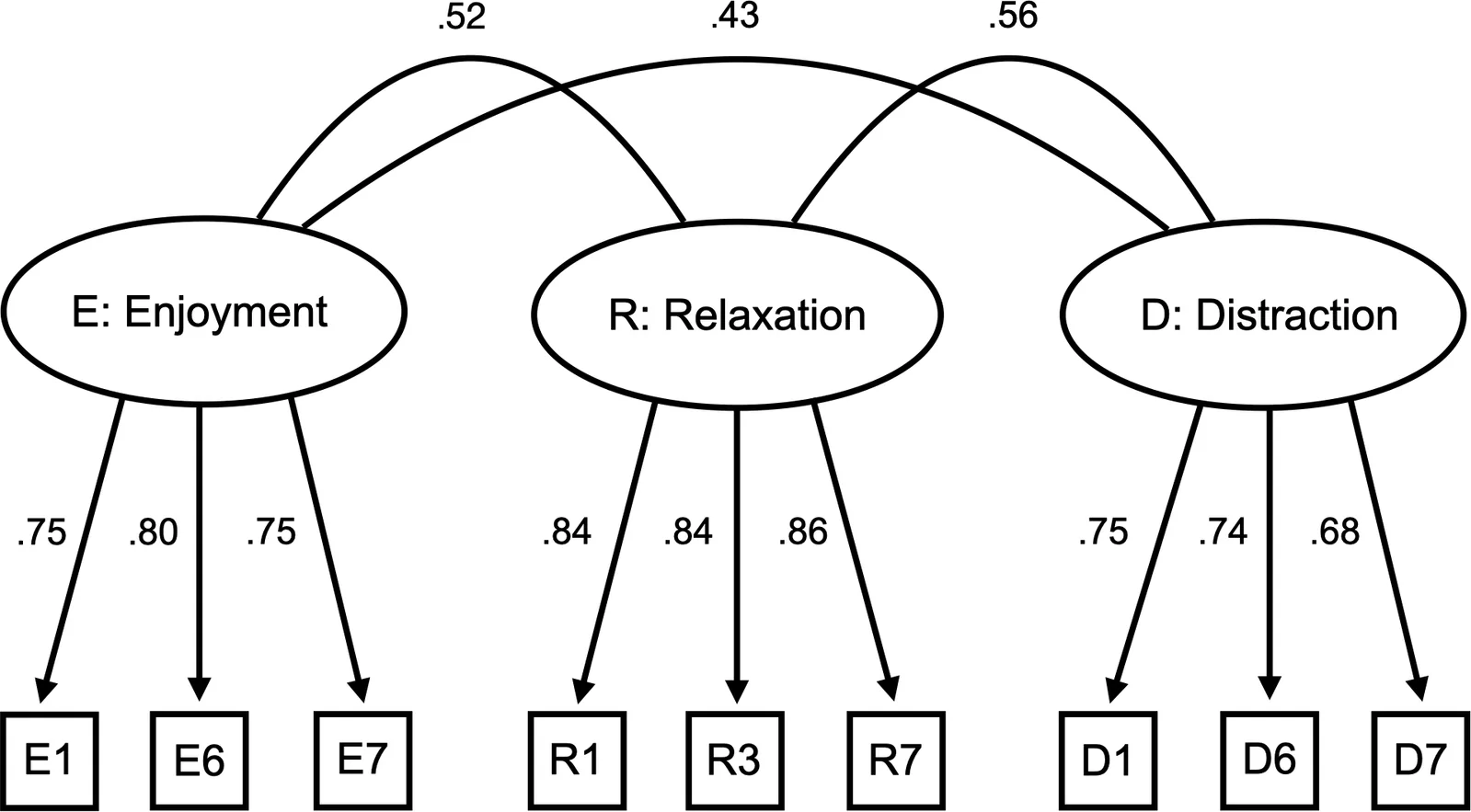
CFA structure of the Enjoyment–Distraction–Relaxation scale. Showing standardised loadings between the three latent factors—Enjoyment, Relaxation, and Distraction—and nine items. For item contents, see S3. Appendix 3.

#### 2.2.2 Scale for Focus–Motivation (FM).

The scale for Focus–Motivation (FM) consists of two sub-scales with each represented by three items. The standardised loadings of the CFA model for FM is shown in Fig 3 but the means and standard deviations of ratings are found in S3 Appendix 3. The model provided an excellent fit to the data ($\chi^{2}$=20.56, *df* = 16, *p* = .196, TLI = 0.987, CFI = 0.993, RMSEA = 0.053, SRMR = 0.033) on all metrics. The items for the motivation and focus sub-scales were equally relevant with both sets of items obtaining high positive loadings within their respective latent factors. This scale passes the metric invariance test (*p* = .55) but fails the scalar invariance test (*p* < .01).

**Fig 3:**
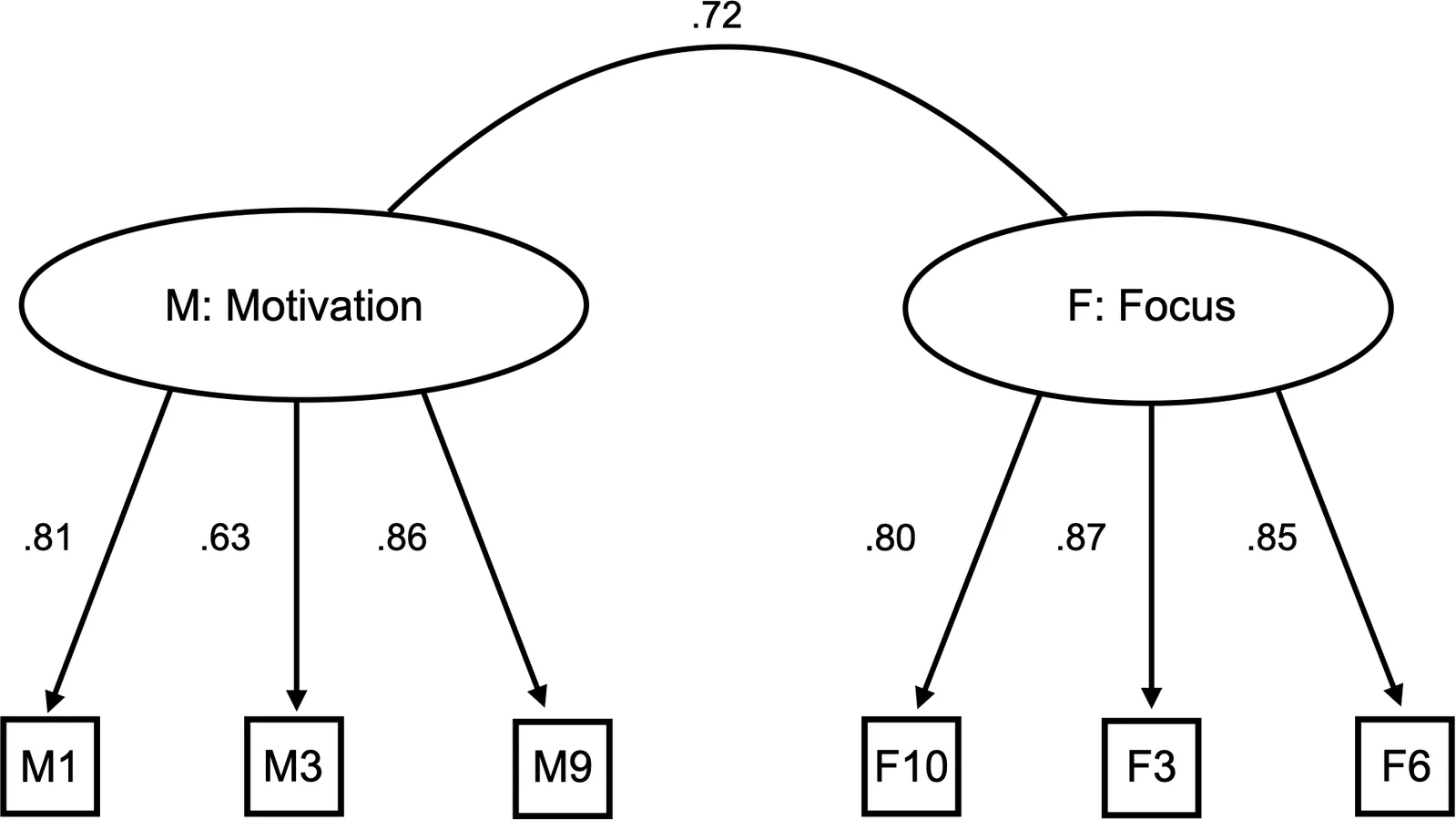
CFA structure of the Focus–Motivation scale. Showing standardised loadings between the two latent factors—Motivation and Focus—and six items.

#### 2.2.3 Scale for Connection–Belonging (CB).

The Connection–Belonging (CB) scale consists of two latent factors, each represented by 3 items with sub-scales labeled as Group bonding (G) and reducing Loneliness (L). Fig 4 suggests that the standardized loadings indicate the correct items being highly loaded on each latent factor. The mode fit is excellent on all fit metrics ($\chi^{2}$=22.14, *df* = 16, *p* = .139, TLI = 0.978, CFI = 0.989, RMSEA = 0.062, SRMR = 0.040). The CB scale also passed the metric invariance test (*p* = .928) and passed the scalar invariance test (*p* = .289).

**Fig 4:**
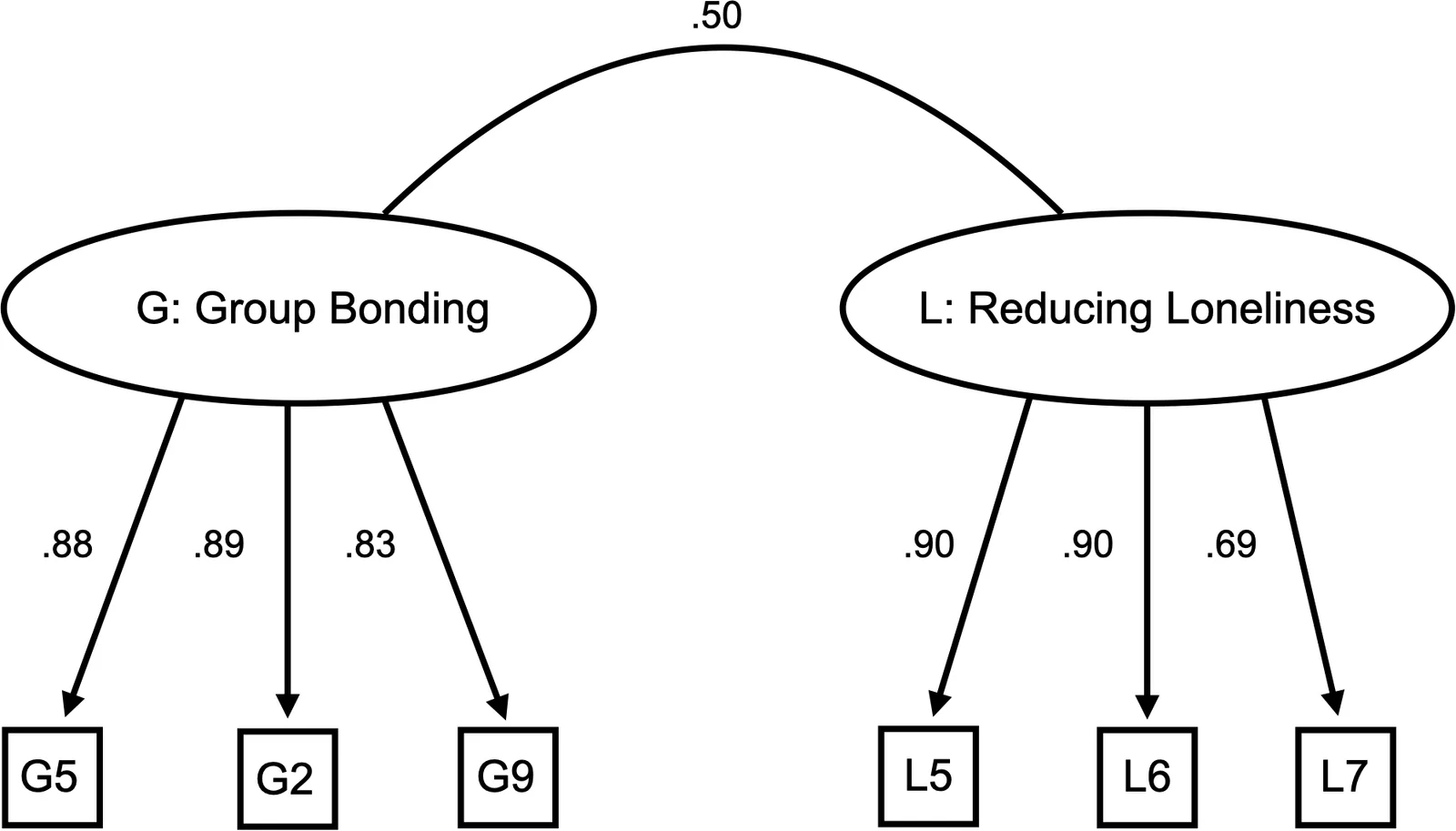
CFA structure of the Connection–Belonging scale. Showing standardised loadings between the two latent factors—Group Bonding and Reducing Loneliness—and six items.

#### 2.2.4 Scale for Personal Emotion Processing (PEP).

The scale for Personal Emotion Processing contains two latent factors Expression (X) and Comforting (C), each represented by three items that loaded clearly onto appropriate factors (see Fig 5). Model fit is very good ($\chi^{2}$=26.87, *df* = 16, *p* = .043, TLI = 0.961, CFI = 0.979, RMSEA = 0.082, SRMR = 0.040) although the absolute fit is borderline acceptable ($\chi^{2}$ should provide a fit above *p* < .05) and RMSEA value exceeds the suggested threshold of 0.08). Considering the high comparative fit indices (CFI and TLI) and the other absolute fit index (SRMR) being very good and under the recommended values, we can interpret the model as providing a good fit. The PEP scale passed the metric invariance test (*p* = .279) but failed to pass the scalar invariance test (*p* < .01).

**Fig 5:**
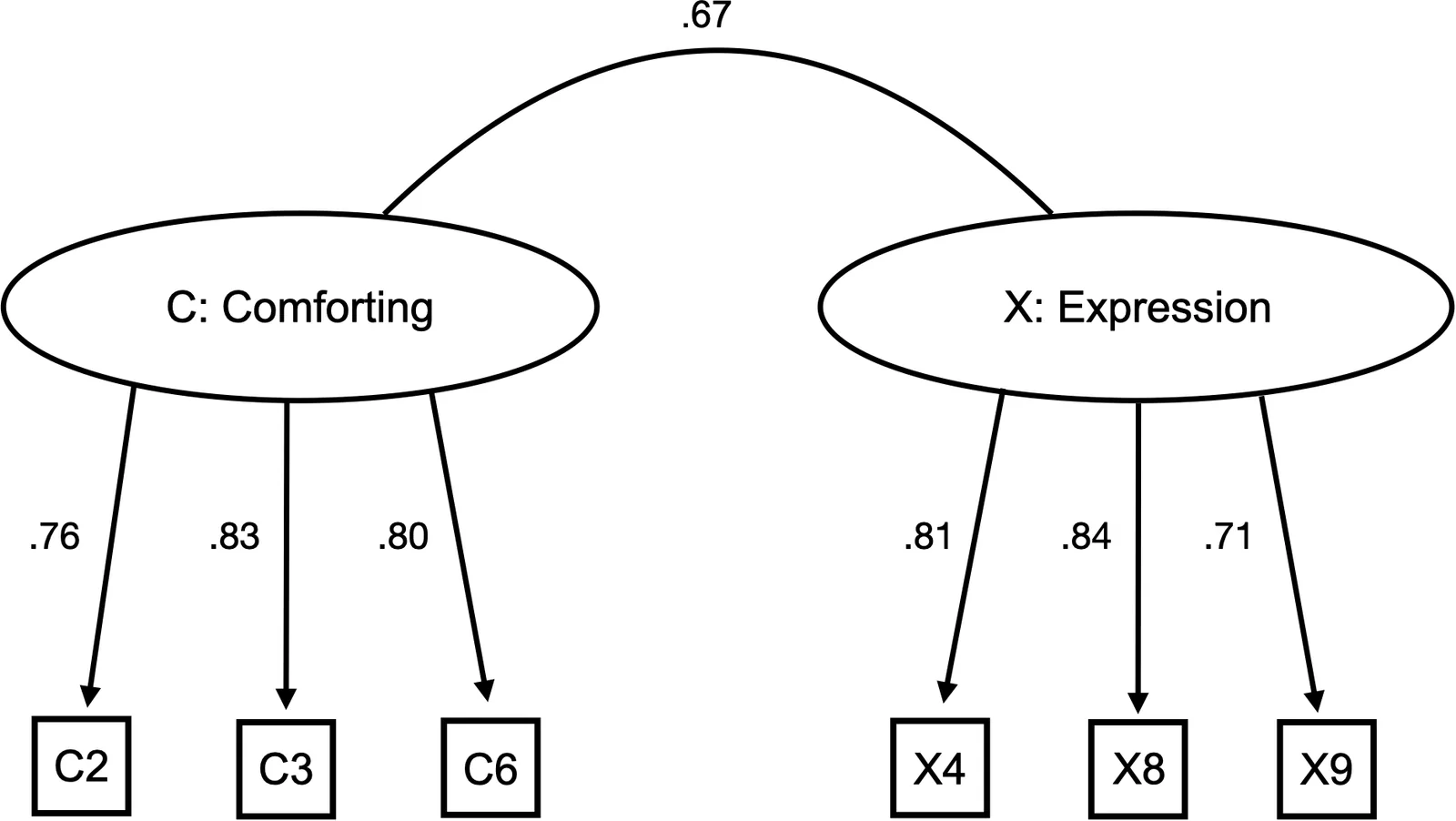
CFA structure of the Personal–Emotion–Processing scale. Showing standardised loadings between the two latent factors—Comforting and Expression—and six items.

#### 2.2.5 Scale for Aesthetic–Interest–Awe (AIA).

The scale for Aesthetic–Interest–Awe consists of three latent factors, with one consisting of two items and the other two having 3 items. The sub-scales were labeled Spirituality (S), Curiosity (I), and Beauty (B). Looking at the CFA model output (Fig 6 for standardised loadings), we observe healthy positive loadings for the Spirituality, Curiosity, and Beauty sub-scales. Overall, the CFA model yields a very good fit ($\chi^{2}$=80.10, *df* = 51, *p* = .006, TLI = 0.961, CFI = 0.976, RMSEA = 0.076, SRMR = 0.046), even though the absolute fit index ($\chi^{2}$) fails to support the model. This seems to be due to just 2 items representing the Curiosity sub-scale (I1 and I2) and the poorer fit of the item B5. Additionally, the Aesthetic–Interest–Awe scale fails to pass the metric invariance test (*p* = .022) and also fails to pass the scalar invariance test (*p* < .01). Looking at the loading structure in detail, it is particularly the item B5 that causes minimal issues to the model. However, there is no major gain from eliminating this item from the model to only obtain metric invariance.

**Fig 6:**
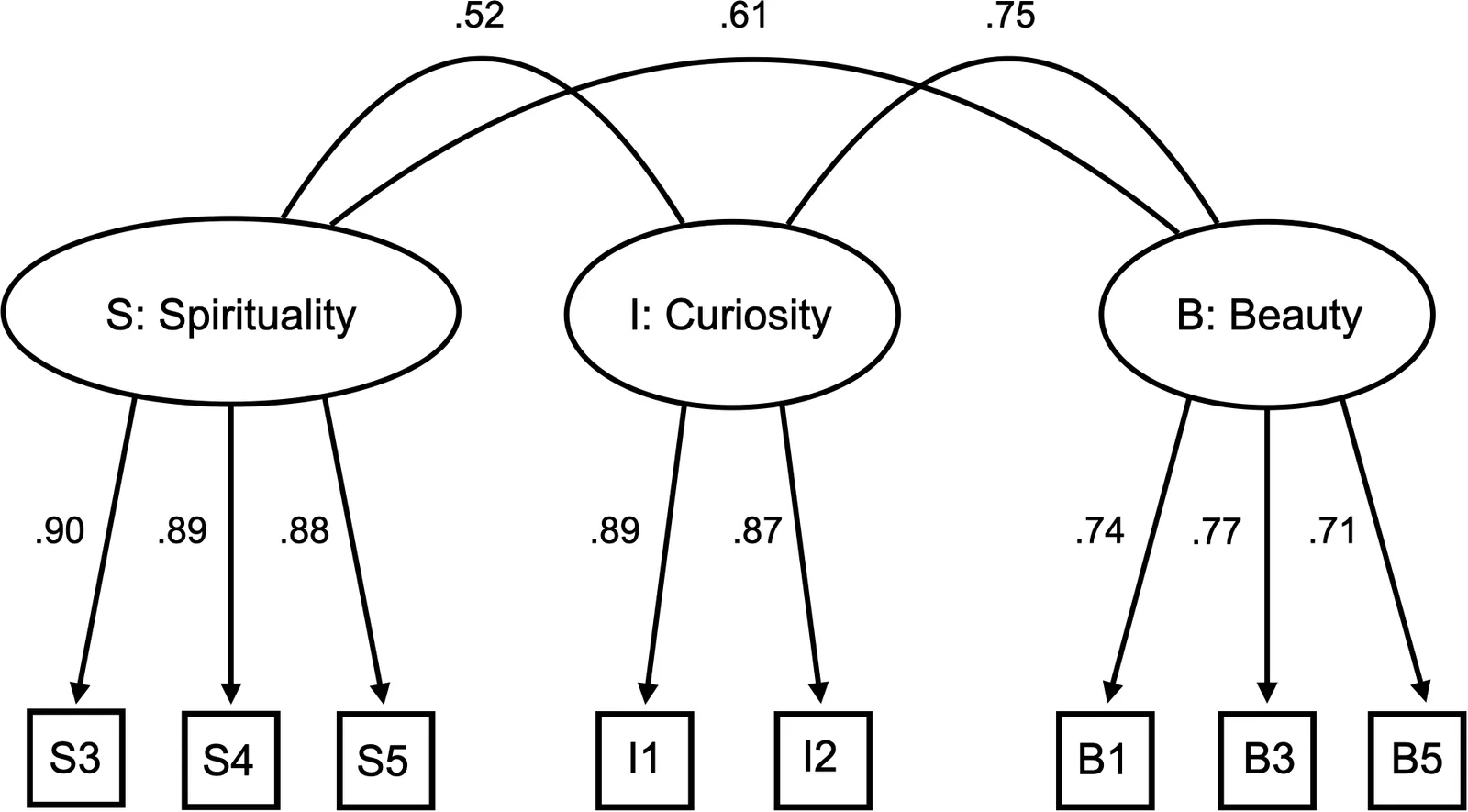
CFA structure of the Aesthetic–Interest–Awe scale. Showing standardised loadings between the three latent factors—Spirituality, Curiosity, and Beauty—and eight items.

### 2.3 Summary

The CFA results with 1200 participants largely corroborate the factor structures for all five scales identified in Experiment 1. The fit indices of each analysis of the scale suggested that no substantial changes to the structure are needed, although we altered the names of the sub-constructs to better reflect the meaning of the items. Absolute fit indices, including $\chi^{2}$, RMSEA, and SRMR, generally indicate acceptable model fit. The only partial exceptions are PEP and AIA, which show borderline fit on some indices. For PEP, $\chi^{2}$ and RMSEA suggest mediocre fit, whereas SRMR, TLI, and CFI exceed conventional thresholds (0.040, 0.95, and 0.95, respectively). Given the well-known sensitivity of the $\chi^{2}$ test to sample size, and the fact that the majority of fit indices indicate good fit, the model for each of the five constructs are considered to provide a reasonable approximation of the data. For AIA, the $\chi^{2}$ test indicates lack of fit, while all other indices suggest acceptable to good fit. This discrepancy may reflect limitations associated with the inclusion of a two item sub-scale for this scale. Because the misfit is confined to only the $\chi^{2}$ statistic, the overall model is likewise deemed to provide an adequate representation of the data. While not optimal, retaining the two item factor (Curiosity) is empirically supported by the model fit. Although, future work should seek to reevaluate the content of the AIA scale and replicate the dimensionality investigation we performed in this article.

As for the measurement invariance testing across the five scales, the models all captured variations between vignettes with the same factor structure (i.e., configural variance). When we also constrained the factor loadings to be equal across the vignettes we observed that all of the scales, except AIA, pass this test (i.e., metric invariance). Only one of the scales passed the last measurement invariance test we performed (Connection-Belonging) which stipulates equal factor loadings and intercepts across groups (i.e., scalar invariance). In practical terms, the results of our measurement invariance testing showcased that the items relate to the latent constructs to a comparable fashion allowing future comparisons of relationships among latent variables. This suggests that scales are similar across vignettes, although the failure to achieve scalar invariance indicates that response patterns or item intercepts differ systematically across vignettes, meaning direct comparisons of latent mean scores between vignettes should be interpreted with caution. Our results do suggest, however, that the items are reflective of their construct regardless of the vignette presented, such that the latent structure does not change between conditions.

#### 2.3.1 Discriminant validity and reliability of the scales.

Moving on to further questions about the structures identified for each of the scales, one can ask five questions about the structures identified: (1) is there an overall hierarchical structure that characterises all of the scales together or is it multidimensional, (2) do the sub-scales provide good discriminant validity within the five scales, (3) will the five scales themselves offer discriminant validity or significant overlap between them, (4) how consistent are the ratings within the sub-scales, (5) is there an indication of convergent validity such as an interpretable relationship between the scales and emotions?

To provide an answer to the first question, we applied two broader structural equation models that we call first order and second order models and applied them to the full data. The first order model contains all 12 sub-scales as a hierarchy under some ambiguous single latent variable. The second order model adds a hierarchical structure to the first order model, where the 12 sub-scales are connected to five scales in a theoretically appropriate fashion (e.g., EDR is linked to E, D, R latent factors, which again are connected to 9 items). While the Episode Model does not propose such a hierarchical structure under a single unifying construct, it was important to review these models to determine whether the structure is better described as multidimensional. We apply these two models to the data using CFA using a robust maximum likelihood estimator (MLR) but without a separate grouping variable for vignettes. The model fit for the first order model is relatively good ($\chi^{2}$=1617.75, *df* = 494, *p* < .001, TLI = 0.949, CFI = 0.958, RMSEA = 0.044, SRMR = 0.039) despite exceeding one absolute fit criteria ($\chi^{2}$). The second order model, however, receives clearly poorer fit ($\chi^{2}$=2512.26, *df* = 538, *p* < .001, TLI = 0.918, CFI = 0.926, RMSEA = 0.055, SRMR = 0.060), and the second order model was inferior when compared to the first order model ($\Delta \chi^{2}$(44)=689.1, p < .001). For this reason, it is not feasible to portray the model as a single, hierarchical instrument that contains five scales each having 2–3 sub-scales to capture all variance. Various other formulations (bifactor model, second-order only, first-, second- and generic third-order factor) were tried but all received poor fit indices. It seems that the 12 separate sub-scales are able to portray the underlying covariance structure of the data adequately but forcing these 12 latent factors also into 5 latent structures under a single hierarchical model is a step too far. It is better to view each of the five scales as their own separate latent variables—closely related but capturing distinct aspects of an emotional episode—and judge each by their own model fit. As such, it should be assumed that the latent structure is multidimensional rather than any hierarchical structure constrained to any single overarching latent variable. Closer scrutiny of coefficients for both models suggest that not all 12 sub-scales perfectly align themselves under just one higher level construct as some of the sub-scales correlate to a degree in these hierarchical models. This leads to the second question about what structures were identified for the five separate scales.

Next, we applied discriminant validity to the scales, and used the updated criteria from Rönkkö and Cho [75] for nested mode comparison to check whether latent variable correlations are sufficiently low so that the sub-constructs can be considered to represent distinct constructs. This calculation was done using additional functionality to lavaan library added by SemTools [76]. Table 1 demonstrates the overlap of the sub-scales within the five scales. All nine pairwise comparisons yielded significant chi-square differences ($\Delta \chi^{2}$ range: 21.1–189.9, all p < .001), indicating that no two factors could be collapsed into a single latent factor. Even though one scale, namely PEP, receives high interfactor correlation (.745 [.609–.881]), this is considered sufficiently differentiated, and the rest are firmly between .32 and .70. These results collectively suggest that sub-constructs within each scale are sufficiently discriminable from each other.

**Table 1:** Discriminant validity of sub-constructs within each scale.

Construct	Subcons	est	ci.lower	ci.upper	Df	$\Delta \chi^{2}$	P
EDR	E D	0.330	0.166	0.494	26	110.6	<.001
EDR	E R	0.493	0.343	0.643	26	189.9	<.001
EDR	D R	0.593	0.467	0.719	26	71.0	<.001
FM	F M	0.697	0.543	0.850	9	26.4	<.001
CB	G L	0.138	−0.049	0.325	9	95.4	<.001
PEP	C X	0.745	0.609	0.881	9	21.1	<.001
AIA	S I	0.324	0.186	0.462	19	92.4	<.001
AIA	S B	0.596	0.493	0.699	19	70.3	<.001
AIA	I B	0.522	0.385	0.658	19	62.9	<.001

As a variant of discriminant validity, we also obtained Heterotrait-Monotrait Ratio (HTMT [77]; sometimes called *disattenuated correlation using parallel reliability*, see [75]) scores across sub-scales as HTMT has been shown to be less biased estimator of discriminant validity than McDonald’s ω or the $\chi^{2}$ comparisons [78]. We calculated HTMT ratios for sub-scales (shown in Table 2), which yielded a range of values between 0.272 to 0.809 with a median of 0.484. As the HTMT values under .85 indicate adequate discriminant validity [77], and hence the sub-scales do not significantly overlap. To test the discriminant validity between the five scales, we would need the second order model to have provided a good starting point for the model. As the model fit was poor, exploring the discriminant validity at the level of these high level constructs is not really recommended, but just for the sake of comparison, calculating the HTMT ratios for the five scales produced values between 0.459 and 0.871 with a median of 0.692. The highest HTMT value was observed between PEP and EDR (0.871), suggesting similarities in item wordings and potential overlap between these two scales. However, this interpretation should be qualified by the caveat that the underlying structural model is intended as a diagnostic tool rather than a reliable representation of the data.

**Table 2:** Overlap between all sub-scales (HTMT values).

	E	D	R	M	F	G	L	C	X	S	I
**D**	0.443										
**R**	0.521	0.552									
**M**	0.515	0.459	0.272								
**F**	0.396	0.426	0.405	0.721							
**G**	0.525	0.276	0.309	0.346	0.280						
**L**	0.463	0.654	0.494	0.424	0.444	0.514					
**C**	0.563	0.745	0.76	0.401	0.464	0.432	0.714				
**X**	0.542	0.654	0.474	0.597	0.497	0.568	0.631	0.682			
**S**	0.338	0.458	0.429	0.299	0.315	0.451	0.472	0.534	0.532		
**I**	0.528	0.358	0.388	0.405	0.379	0.552	0.46	0.459	0.536	0.517	
**B**	0.809	0.431	0.577	0.394	0.367	0.633	0.517	0.688	0.619	0.618	0.758

*Note.* E = Enjoyment, D = Distraction, R = Relaxation, M = Motivation, F = Focus, G = Group bonding, L = Loneliness reduction, C = Comforting, X = Expression, S = Spirituality, I = Curiosity, and B = Beauty.

To calculate reliability (composite reliability) for all sub-scales, we use the first-order model to calculate reliability and discriminant validity indices. This allowed us to calculate the composite reliability scores (for McDonald’s ω see [79]; see also [80]) for all 12 sub-scales simultaneously using semTools [76]. The ω scores range from 0.769 [CI 0.739–0.794] (D) to 0.903 [CI 0.891–0.915] (C), and all but two (D, B) provide good reliability (ω>0.80) and those two (D ω = 0.769 [CI 0.739–0.794] and B ω = 0.783 [CI 0.761–0.804]), acceptable reliability.

#### 2.3.2 Establishing convergent validity for future application.

To assess preliminary convergent validity of the MEEM scales, we examined emotion ratings associated with each vignette. While there are no directly comparable state based measures for the same constructs, establishing this comparison between common emotion rating schemes will be useful for subsequent applications. Participants rated the emotions experienced in response to each vignette—representing a typical situation—using the GEMIAC checklist and the HAAS scale. To relate the sub-scale structure to the HAAS framework, we employed the first-order model and correlated individual-level sub-scale scores with HAAS adjective ratings aggregated into four affective quadrants (positive valence–high arousal, positive valence–low arousal, negative valence–high arousal, and negative valence–low arousal). For GEMIAC, we identified the three most frequently endorsed emotions for each sub-scale.

The results, presented in Table 3, suggest that most sub-scales occupy distinct locations within the affective circumplex, with correlation patterns that align intuitively with their conceptual meanings. For example, Enjoyment is positively correlated with positive-valence emotions and negatively correlated with negative-valence emotions, and is primarily associated with higher arousal. In contrast, Distraction is less positively valenced and shows small but positive correlations with negative-valence quadrants (*r* = 0.12 and 0.14 for high- and low-arousal negative valence, respectively). Relaxation shows strong associations with positive-valence, low-arousal emotions, as expected. More comparisons, than what we are able to perform here, will be needed across a variety of real-world applications, methods, and comparable instruments to faithfully review the convergent validity between MEEM and affectual states.

**Table 3:** Correlation between factor scores and emotion quadrants (from HAAS) and the three most frequently nominated emotions from GEMIAC checklist.

Sub-scale	Positive		Negative		Top 3 GEMIAC emotions
	High	Low	High	Low	
Enjoyment (E)	0.56	0.45	−0.30	−0.27	Energetic, lively; Joyful, wanting to dance; Inspired, enthusiastic
Distraction (D)	0.31	0.31	0.12	0.14	Inspired, enthusiastic; Tense, uneasy; Relaxed, peaceful
Relaxation (R)	0.30	0.61	−0.12	−0.05	Relaxed, peaceful; Inspired, enthusiastic; Moved, touched
Motivation (M)	0.55	0.13	0.00	−0.06	Energetic, lively; Powerful, strong; Inspired, enthusiastic
Focus (F)	0.48	0.24	−0.08	−0.13	Inspired, enthusiastic; Relaxed, peaceful; Powerful, strong
Group bonding (G)	0.45	0.25	−0.19	−0.25	Nostalgic, sentimental; Inspired, enthusiastic; Energetic, lively
Loneliness reduction (L)	0.35	0.33	0.00	0.02	Relaxed, peaceful; Full of tenderness, warmhearted; Nostalgic, sentimental
Comforting (C)	0.39	0.48	−0.04	0.01	Melancholic, sad; Agitated, aggressive; Tense, uneasy
Expression (X)	0.50	0.26	0.02	−0.03	Moved, touched; Nostalgic, sentimental; Powerful, strong
Spirituality (S)	0.38	0.35	−0.03	−0.03	Filled with wonder, amazed; Moved, touched; Enchanted, in awe; Filled with wonder, amazed
Curiosity (I)	0.54	0.31	−0.18	−0.24	Moved, touched; Inspired, enthusiastic; Enchanted, in awe
Beauty (B)	0.58	0.50	−0.26	−0.25	Relaxed, peaceful; Moved, touched; Inspired, enthusiastic

However, the HAAS scale does not fully differentiate several sub-scales, as multiple pairings exhibit similar correlation profiles (e.g., Enjoyment and Curiosity; Enjoyment and Group Bonding). The GEMIAC checklist, which includes 15 emotion terms, yielded intuitively interpretable associations with the sub-scales. Across the top three selections for each sub-scale, 13 unique emotions were identified among 32 total nominations, indicating broad affective coverage. The most negatively valenced emotions were associated with Distraction (e.g., tense, uneasy) and Comforting (e.g., melancholic, sad; agitated, aggressive; tense, uneasy). In contrast, Spirituality and Curiosity were associated with a range of emotions commonly linked to aesthetic experiences (e.g., filled with wonder, amazed; moved, touched; enchanted, in awe).

### 2.4 Discussion

We now have 5 scales which capture specific episodes related to music. The analyses clarified the relationships between the sub-scales by demonstrating that within each scale, the sub-scales are sufficiently distinct and even across the scales, there is no substantial overlap between all 12 sub-scales. Also, the data is consistent and reliable within each sub-scale. Despite this first level of success and clarity, the data, however, does not support a hierarchical structure where all five scales with the appropriate sub-constructs combined under a single latent variable would capture the variance in the ratings. Instead, this suggests that the latent structure is multidimensional with five scales that capture distinct aspects but are closely related. This is analogous to other instruments such as Maslach Burnout Inventory (MBI [81]) or Big Five Personality Inventory (BFI [82]) where studies have found support for separate factors but not the hierarchical model (e.g., see [83] for MBI, and [84] for BFI). For this reason we refrain from calling this measure of emotional episodes with music (MEEM) as a single measurement model and prefer five related scales targeting separate functions provided by the theory. Such an interpretation is consistent with the probable use of the scales: to target specific types of episodes. For example, it is unlikely that two contrasting episodes, Focus–Motivation and Aesthetic–Interest–Awe are needed in the same study setup, because these functional uses of music and situations are likely to be entirely different sets of experiences and contexts. Furthermore, such modularity aligns with the original theory, which posits that the episode types can occur simultaneously to a varying degree in a given everyday experience [34]. Separate scales for each episode type allows treating each episode type as a separate dimension and to explore their potential co-occurrence across situations.

#### 2.4.1 Limitations and future directions.

Among other limitations, there are specific limitations related to our methodological decisions and analytical choices. To situate and contextualise the emotional experiences participants had in mind when rating the items, we employed the experimental vignette method (see [51]). However, we used a single vignette for each sub-scale within an episode, whereas a factorial vignette design could have been employed to disentangle and diagnose the effects of different vignette elements [85]. Using vignettes in our experimental design, however, highlights another concern for ecological validity. While we achieved good markers of reliability, generalising the experimental vignette results to real world scenarios should be approached with healthy scepticism. Such practice is common when determining the suitability of an instrument in a particular study design or for a specific purpose. In the short term, those who wish to apply MEEM should seek to review and detail validity evidence along with their primary study aims. The community can build upon the validity evidence provided here, by investigating different real-world scenarios, testing effects of administration duration from the episode experience, and assessing response patterns between laboratory and ESM or EMA designs. While the limitation is an issue for the present work, in the future meta reviews of studies using MEEM will assist in developing informed guidelines for administration in a wide array of ecological settings.

In our analyses, we adopted a confirmatory approach to factor analysis (confirmatory factor analysis, CFA), as the items and constructs were informed by two rounds of expert evaluation, including rankings of item relevance for each sub-construct [36]. A more traditional approach would have been to conduct an exploratory factor analysis (EFA) followed by CFA. However, given the extensive prior knowledge of the items and constructs, our primary aim in the first experiment was to evaluate whether the observed factor structure was consistent with theoretical predictions and to select the best-performing items to represent each factor [44,46]. For that reason, we chose an exploratory CFA approach in the first experiment to 1) assess whether our assumed structure from the theory (see [34]) and content validity (see [36]) was coherent with the data we gathered here, and 2) to explore models with different collections of items, in our case we kept this simple and only took the top three items from each sub-construct.

In Experiment 1, we observed a clear reduction in conceptual breadth from the large pool of items identified by experts to a smaller set of items that best captured the latent factors [36]. Although such reduction is often necessary when developing scales with relatively few items [58], this narrowing process may introduce construct drift and thus pose a risk to instrument validity. To account for such issues, we retested all of the scales with a new sample of participants in Experiment 2. Again, we employed CFA but our use of the technique in Experiment 2 followed a more conventional confirmatory approach that is common for establishing whether the structure uncovered in an explored model can be replicated [44,46]. In light of alternative statistical approaches, such as network analysis [86], the limitation of our approach is that it is a preliminary assessment of the structure under these experimental conditions. Future work should build upon the development of the MEEM scales to test causal systems with such network approaches like temporal time series [87]. Nevertheless, the CFA approach we chose has the advantage of producing a focused and optimised instrument upon which future work can further investigate validity and reliability.

In Experiment 2, we conducted CFA to corroborate the factor structure using an independent dataset. One specific limitation concerns interpreting the two item sub-scale (Curiosity, see S2 Appendix 2). While we mitigated potential issues with identification by adding supplementary items, those who wish to use the AIA module should seek to add another indicator for the Curiosity sub-scale. Our recommended best option would be the third highest loading item from Exp. 1 (I3, see S2 Appendix 2) as a candidate to reinvestigate and mitigate potential identification issues. At this stage, we also examined convergent validity using two external measures (HAAS and GEMIAC). Although it would be informative to extend the convergent validity to instruments that capture a wider range of functional uses of music (e.g., the Healthy – Unhealthy Music scale [15]; Brief Music in Mood Regulation scale [17]; Music Use and Background Questionnaire [10]; Barcelona Music Reward Questionnaire [12]), all of these instruments assess general, trait-like tendencies rather than experiences tied to specific situations or contexts. None of the trait based measures would be suitable to provide validity evidence for states [39,88]. A limitation of the present development process concerns the lack of preexisting state-based instruments which can serve as a benchmark in the particular topic area. In particular, our review of convergent validity lacks the necessary comparisons with instruments that capture similar constructs, let alone a diverse array of methods. Longitudinal studies using MEEM could provide a similar trait assessment to compare with common trait measures. This would not address the emergence of particular states in dynamic and contextualised situations nor provide a strong validity or reliability assessment for the state based purpose.

A useful direction for future research would be to examine the descriptive schemes associated with the theoretical model in terms of their constituent components, potentially using a factorial allocation of these components. These descriptive schemes encompass central aspects such as listening modes, attention, and musical meaning (see [34]), which may provide more concrete conceptual handles for specifying and understanding the underlying drivers of emotional experiences associated with music. A related avenue for comparison, that could offer a potentially informative perspective for convergent validity, is using the BRECVEMAC framework to assess multiple features and mechanisms at the same time [22,89]. Although BRECVEMAC is a theoretical framework rather than a psychometrically validated instrument, it provides a useful conceptual account of how surface features of music may give rise to emotional responses. Such work, however, would require a congealing of different epistemic perspectives to categorise the emotions observed through a methodological framework that captures the dynamic, procedural, emotional episodes people have within musical situations.

Finally, we acknowledge the limitations of our samples. All participants were based in the UK, were English speakers, and therefore represent a Western, educated, industrialised, rich, and democratic (WEIRD [90]) population. Although the sample departs from typical student samples in terms of age (M = 45 years) and exhibits a relatively balanced gender distribution, these characteristics do not mitigate the broader limitations regarding cultural representativeness. Substantial further work is required to examine the extent to which the five proposed scales and their measurement generalise across cultural contexts. We anticipate variation both within Western societies and, more markedly, when the instrument is applied in non-Western settings. While the theoretical framework was developed with the intention of integrating functions of music that have been discussed across diverse cultural literatures, we emphasise that the current operationalisation, as well as its construct validity, should be regarded as provisional outside the present cultural context and will require re-evaluation, adaptation, and potentially reconceptualisation in other cultural regions.

## 3 Conclusions

The purpose of the present research was to evaluate a set of constructs and items designed to elucidate emotional experiences related to music. Guided by theory and subsequent content-validity procedures [34,36], we assessed and refined an instrument comprising one scale for each of the five postulated constructs, with each construct further differentiated by two to three sub-constructs. The labelling of these sub-constructs evolved slightly during the development process, as empirical results indicated that the best-fitting items emphasised more specific facets of the sub-constructs than what was assumed following the prior content validity procedure [36]. Across the two experiments, empirical evidence provided strong support for the overall proposed sub-construct structure for each scale. This was reflected in good CFA model fits to the data from Experiment 2, with clear differentiation between the sub-constructs within the five scales, and initial evidence of construct validity, as indicated by systematic associations between the five scales and two distinct types of emotion ratings. We label this instrument as the Measure of Emotional Episodes with Music (MEEM).

MEEM is a modular tool, comprising five distinct scales, designed to capture the functional use and affect regulatory goals people attribute to music within diverse contexts and situations. This multidimensional interpretation aligns with theoretical assumptions of the Episode Model [34]. Our exploration of a hierarchical model with a specified single variable was not supported. To date, no existing instrument explicitly operationalises the situational or contextual aspects of emotional experiences with music. Although prior work has offered empirical observations [91] and theoretical accounts of musical contexts [32,33,92], these have not been developed into a dedicated instrument. Importantly, MEEM is intended to capture states (emotional experiences as processes grounded in situations and fluctuating psychological functions) rather than stable traits (and tendencies) that have been previously captured by music-related instruments (e.g., B-MMR [17]; HUMS [15]). Now, MEEM offers researchers new avenues to observe the emergence of emotions through how people change their perception of the music within the situation based on their goals or what function they believe the music has for them in a present moment.

A key strength of MEEM lies not only in its rigorous theory-driven development and adherence to established scale-construction procedures, but also in its modular and adaptable design. The instrument can be administered in full or by selecting individual scales to match specific research or applied use cases. For example, studies focusing on music for relaxation may primarily employ the Enjoyment–Distraction–Relaxation scale, whereas research on music in sports or exercise contexts may prioritise Focus–Motivation, while still allowing for the inclusion of additional scales to capture secondary emotion-related processes. Taken together, MEEM provides a flexible framework for advancing the empirical study of situational emotional experiences with music and offers a foundation for future refinement, validation, and cross-contextual application.

## Supporting information

S1 Appendix Vignettes. (PDF)

S2 Appendix Factor Loadings for 87 items. (PDF)

S3 Appendix Item Statistics for 35 items. (PDF)

S4 File MEEM instrument. (PDF)
