# Supplementary material for: Measure of Emotional Episodes with Music (MEEM): Development and psychometric evaluation of a modular instrument
Source: PLoS One. 2026 Oct 1;21(10):e0359288. doi: 10.1371/journal.pone.0359288 (PMC13630252; doi:10.1371/journal.pone.0359288)
Supplement: Supplementary file 1 [file pone.0359288.s001.pdf]

Appendix 1

EDR vignettes

Enjoyment (E): "*You are attending a celebration where music is the heart of the party. You are able to join in the fun which is aided by the music throughout the event".*

Distraction (D): "*It was pointed out that you missed an important detail in your work which you now have to redo. You put on a music playlist to drown out self-criticism as you fix the mistake".*

Relaxation (R): "*After a long and demanding week, you take time to rest by listening to music. The stress melts away and you start to recover as you listen along".*

CB vignettes

Group Bonding (G): "*While with your friends at a cafe, a particular song comes on. You instantly recognise the music from your past, leading to an exchange of stories about your experiences together"*.

Reducing Loneliness (L): "*As you travel alone for an extended period, you put on your partner's favourite music. The music makes you feel as if they are there with you"*.

FM vignettes

Focus (F): "*You have an intricate task which requires your attention. To put your mind to it, you turn on a specific music playlist"*.

Motivation (M): "*In order to get ready to exercise you decide to put on music which gives you the right frame of mind"*.

PEP vignettes

Comforting (C): "*You've just been told that you didn't get the job you applied for. To handle the news, you turn to music to provide a sense of support as you manage your thoughts*".

Expression (X): *"While listening to a playlist, you come across a song that deeply resonates with what you are experiencing. It communicated something about yourself that you've struggled to explain to others*".

AIA vignettes

Spirituality (S): *"Halfway through a concert, you realise you have been fully absorbed in the power and meaning of the music. It feels as if the music has taken you into a transcendent experience, which is exactly what you hoped for before the concert*".

Curiosity (I): *"While out for a walk, you hear a busker playing incredible sounding music you have never discovered before. You are not sure what is attracting your interest to the music but you allow yourself to be taken in by the music and its new emotional direction*".

Beauty (B): *"As you sit outside on your lunch break, you hear amazing music drifting in from a nearby building. While admiring the graceful nature of the music itself, you notice how it intensifies your lunch break into a unique and lasting experience*".
