# Supplementary material for: Measure of Emotional Episodes with Music (MEEM): Development and psychometric evaluation of a modular instrument
Source: PLoS One. 2026 Oct 1;21(10):e0359288. doi: 10.1371/journal.pone.0359288 (PMC13630252; doi:10.1371/journal.pone.0359288)
Supplement: Supplementary file 2 [file pone.0359288.s002.pdf]

Appendix 2

Table 1. CFA results for the 87 items used across the five sub-experiments

Item	Item ID	Mean (SD)	Factor 1	Factor 2	Factor 3
EDR					
The music made me feel calm or relaxed	**R1**	3.91 (0.99)	**0.794**	0.241	0.311
I used the music to soothe my mind	R2	3.88 (1.01)	**0.660**	0.229	0.482
I was using the music to relax	**R3**	3.94 (1.04)	**0.776**	0.176	0.312
I used the music to calm myself down and relax	R4	3.92 (1.02)	**0.780**	0.220	0.359
Listening to the music helped me recharge	R5	3.90 (1.00)	**0.546**	0.388	0.310
Relaxing with the music made me feel better	R6	4.09 (0.95)	**0.720**	0.372	0.268
Music provided a relaxing mood	**R7**	3.99 (0.99)	**0.760**	0.339	0.275
Listening to music was a good way of entertaining myself	**E1**	4.09 (0.93)	0.143	**0.791**	
The music gave me pleasure	E2	4.23 (0.87)	0.382	**0.764**	
I enjoyed how the music made me feel	E3	4.19 (0.89)	0.377	**0.725**	0.234
I was delighted by the music	E4	4.01 (0.98)	0.253	**0.780**	0.236
I felt like I was enjoying myself because of the music	E5	4.01 (0.97)	0.182	**0.807**	0.190
I was having a good time listening to the music	**E6**	4.12 (0.97)	0.220	**0.844**	0.196
I used the music to have a good time	**E7**	3.91 (1.09)		**0.811**	
The music helped me break away from the situation	**D1**	3.69 (1.10)	0.257	0.227	**0.741**
The music pushed my worries aside	D2	3.72 (0.99)	0.453	0.198	**0.641**
The music allowed me to escape from my daily routines	D3	3.75 (1.01)	0.164	0.325	**0.671**

Music distracted my mind from the outside world	D4	3.90 (0.95)	0.363	0.232	**0.689**
I wanted to have background music on to distract myself	D5	3.64 (1.11)	0.255		**0.710**
Music acted as a mental shield against my distressing thoughts	**D6**	3.55 (1.16)	0.292		**0.745**
I wanted the music to help me forget what I was doing	**D7**	3.45 (1.13)	0.125		**0.774**
The music made me forget what I was thinking about	D8	3.63 (1.01)	0.299	0.194	**0.699**
Cumulative Variance			0.688	0.239	0.469
CB					
I felt like a member of a group while listening to the music	G1	3.41 (1.24)	**0.780**	0.143	
I felt a bond with the other people listening to the music	**G2**	3.75 (1.09)	**0.844**	0.177	
The music made me feel more connected with those around me	G3	3.63 (1.14)	**0.797**	0.112	
I felt more connected with other people because of the music	G4	3.85 (1.04)	**0.758**	0.318	
I felt connected to the people I was sharing this musical experience with	**G5**	3.78 (1.01)	**0.850**	0.141	
I felt connected with people who also listen to this music	G6	3.87 (1.01)	**0.668**	0.371	
The music made me feel connected to my peers or family	G7	3.95 (1.01)	**0.614**	0.469	
The music brought people together	G8	3.75 (1.18)	**0.716**	0.243	
The music made me feel more connected to a group	**G9**	3.58 (1.15)	**0.806**	0.168	
The music helped me feel closer to people	L1	3.93 (0.92)	**0.686**	0.479	
The music made me feel connected to the people I care about	L2	4.17 (0.91)	0.574	0.513	
The music made me feel like I belonged	L3	3.69 (1.04)	**0.701**	0.378	

I felt a special connection to the people I shared this music with	L4	3.96 (0.99)	**0.744**	0.226
Listening to music made me feel less lonely	**L5**	3.77 (1.15)	0.214	**0.882**
The music helped me feel less lonely	**L6**	3.75 (1.14)	0.195	**0.888**
I used the music to keep me company	**L7**	3.66 (1.26)	0.140	**0.840**
Cumulative Variance			0.449	0.673
FM				
The music was motivating me to do something	**M1**	3.995 (1.24)	0.307	**0.844**
Listening to music made me more alert	M2	3.75 (1.11)	0.523	0.539
The music pumped me up	**M3**	3.97 (1.23)	0.196	**0.849**
I used the music to motivate myself	**F1 / M9**	4.0 (1.11)	0.305	**0.869**
The music kept me concentrated on the task I was supposed to do	F2	3.86 (1.00)	**0.812**	0.306
The music helped me concentrate	**F3**	3.79 (1.05)	**0.862**	0.289
I chose to listen to music to help me focus	F4	3.9 (1.04)	**0.821**	0.321
The music helped me accomplish work I needed to do	F5	3.88 (0.96)	**0.698**	0.519
Music helped me focus on what I was doing	**F6**	3.86 (1.06)	**0.868**	0.306
The music motivated me to finish the task I had to do	F7	3.91 (1.05)	0.480	**0.724**
The music reduced my motivation	F8	1.56 (0.86)		
I used the music to block out distractions	F9	3.56 (1.24)	**0.702**	0.194
I was more focused while listening to the music	**F10**	3.79 (1.09)	**0.844**	0.305

The music let me focus on the present moment	F11	3.81 (1.05)	**0.730**	0.423
Cumulative Variance			0.415	0.699
PEP				
I used the music to cope with my situation	C1	3.98 (0.91)	0.384	**0.694**
I found solace in the music	**C2**	4.05 (0.86)	0.294	**0.807**
Listening to music comforted me	**C3**	4.20 (0.86)	0.253	**0.805**
The music helped me understand different feelings in myself	C4	3.57 (1.07)	**0.704**	0.309
The music was like therapy for me	C5	3.88 (1.01)	0.445	**0.623**
The music was comforting to me	**C6**	4.23 (0.80)		**0.816**
The music helped me process my emotions	C7	3.93 (0.99)	**0.746**	0.390
I felt comforted by the music, as if it understood me	X1	3.88 (0.98)	0.547	0.539
The music helped me reflect on how I was feeling	X2	4.00 (0.94)	0.594	0.443
The music made me feel secure	X3	3.57 (0.91)	0.295	**0.684**
The music helped me express myself	**X4**	3.69 (1.04)	**0.846**	0.253
The music expressed what I could not	X5	3.71 (1.14)	**0.726**	0.299
Listening to music allowed me to emotionally express myself	X6	3.8 (0.97)	**0.788**	0.332
I used the music to express how I was feeling	X7	3.77 (1.02)	**0.816**	0.332
Listening to music helped me express how I was feeling	**X8**	3.81 (1.02)	**0.841**	0.264
I vented my emotions by listening to music that expressed my feelings	**X9**	3.76 (1.03)	**0.742**	0.295

The music let me vent my frustration	X10	3.6 (1.09)	**0.644**	0.271	
The music helped me work through my thoughts	X11	3.9 (0.86)	0.475	0.604	
The music helped me release my mental tension	X12	4.03 (0.82)	0.385	0.529	
Listening to music allowed me to figure out how I wanted to express myself	X13	3.53 (1.00)	**0.752**	0.363	
Cumulative Variance			0.373	0.644	
AIA					
I was completely immersed in the music, as it altered my state of being	S1	3.53 (1.15)	**0.654**	0.510	0.197
I felt like the music was lifting me into the air	S2	3.04 (1.20)	**0.668**	0.411	0.208
The music provided me with a spiritual experience	**S3**	3.03 (1.30)	**0.857**	0.166	0.190
The music related to my spirituality	**S4**	2.91 (1.28)	**0.878**	0.141	0.226
I wanted the music to take me somewhere spiritually	**S5**	3.01 (1.27)	**0.891**		0.244
I experienced reality differently while listening to the music	S6	3.36 (1.19)	**0.690**	0.410	0.197
Listening to the music stimulated my curiosity	**I1**	3.95 (0.98)	0.238	0.265	**0.847**
The music sparked my curiosity	**I2**	3.95 (0.99)	0.238	0.259	**0.831**
Listening to the music was an intellectual experience for me	I3	3.22 (1.12)	0.438	0.217	0.585
I let go of control to experience new and unfamiliar music	I4	3.41 (1.13)	**0.630**	0.457	0.182
I wanted the music to make me feel something new	I5	3.60 (1.04)	**0.613**	0.285	0.394
I appreciated the beauty of the music	**B1**	4.4 (0.70)	0.163	**0.740**	0.359
I respected the music for its value as a piece of art	B2	4.16 (0.85)	0.136	0.549	0.532

The experience was enhanced by the beauty of the music	**B3**	4.27 (0.80)	0.177	**0.755**	0.396
The music overwhelmed everything else in the situation	B4	3.49 (1.12)	0.502	0.573	0.154
I had an unforgettable experience alongside the music	**B5**	3.62 (1.11)	0.423	**0.663**	0.127
Cumulative Variance			0.327	0.534	0.710
