# Supplementary material for: Measure of Emotional Episodes with Music (MEEM): Development and psychometric evaluation of a modular instrument
Source: PLoS One. 2026 Oct 1;21(10):e0359288. doi: 10.1371/journal.pone.0359288 (PMC13630252; doi:10.1371/journal.pone.0359288)
Supplement: Supplementary file 3 [file pone.0359288.s003.pdf]

Appendix 3

**Table 1.** Best items for the 5 episode constructs and their subconstructs, 35 items in total.

| Construct | Sub Construct | Item Label | Item | M | SD |
|---|---|---|---|---|---|
| EDR | Enjoyment | E1 | Listening to music was a good way of entertaining myself | 4.16 | 0.85 |
| | | E6 | I was having a good time listening to the music | 4.29 | 0.77 |
| | | E7 | I used the music to have a good time | 3.95 | 0.97 |
| | Distraction | D1 | The music helped me break away from the situation | 3.61 | 1.09 |
| | | D6 | Music acted as a mental shield against my distressing thoughts | 3.30 | 1.24 |
| | | D7 | I wanted the music to help me forget what I was doing | 3.25 | 1.18 |
| | Relaxation | R1 | The music made me feel calm or relaxed | 3.89 | 0.92 |
| | | R3 | I was using the music to relax | 3.83 | 1.01 |
| | | R7 | Music provided a relaxing mood | 3.94 | 0.93 |
| FM | Motivation | M1 | The music was motivating me to do something | 3.51 | 1.05 |
| | | M3 | The music pumped me up | 3.62 | 1.07 |
| | | M9 | I used the music to motivate myself | 3.63 | 1.06 |
| | Focus | F3 | The music helped me concentrate | 3.38 | 1.07 |
| | | F6 | Music helped me focus on what I was doing | 3.46 | 1.08 |
| | | F10 | I was more focused while listening to the music | 3.64 | 1.02 |
| CB | Group Bonding | G5 | I felt connected to the people I was sharing this musical experience with | 3.17 | 1.22 |
| | | G2 | I felt a bond with the other people listening to the music | 3.04 | 1.21 |

| | | | | | |
|---|---|---|---|---|---|
| | | G9 | The music made me feel more connected to a group | 3.00 | 1.24 |
| | Loneliness Reduction | L5 | Listening to music made me feel less lonely | 3.43 | 1.17 |
| | | L6 | The music helped me feel less lonely | 3.39 | 1.15 |
| | | L7 | I used the music to keep me company | 3.55 | 1.09 |
| PEP | Comforting | C2 | I found solace in the music | 3.85 | 0.97 |
| | | C3 | Listening to music comforted me | 4.05 | 0.91 |
| | | C6 | The music was comforting to me | 4.06 | 0.87 |
| | Expression | X4 | The music helped me express myself | 3.38 | 1.07 |
| | | X8 | Listening to music helped me express how I was feeling | 3.53 | 1.07 |
| | | X9 | I vented my emotions by listening to music that expressed my feelings | 3.42 | 1.13 |
| AIA | Spirituality | S3 | The music provided me with a spiritual experience | 2.74 | 1.25 |
| | | S4 | The music related to my spirituality | 2.66 | 1.23 |
| | | S5 | I wanted the music to take me somewhere spiritually | 2.81 | 1.28 |
| | Curiosity | I1 | Listening to the music stimulated my curiosity | 3.32 | 1.11 |
| | | I2 | The music sparked my curiosity | 3.27 | 1.13 |
| | Beauty | B1 | I appreciated the beauty of the music | 4.15 | 0.85 |
| | | B3 | The experience was enhanced by the beauty of the music | 3.83 | 1.03 |
| | | B5 | I had an unforgettable experience alongside the music | 3.11 | 1.18 |
