# Supplementary material for: Measure of Emotional Episodes with Music (MEEM): Development and psychometric evaluation of a modular instrument
Source: PLoS One. 2026 Oct 1;21(10):e0359288. doi: 10.1371/journal.pone.0359288 (PMC13630252; doi:10.1371/journal.pone.0359288)
Supplement: Supplementary file 4 [file pone.0359288.s004.pdf]

Measure of Emotional Episodes with Music (MEEM)

Connor Kirts, Suvi Saarikallio, Tuomas Eerola

Overview: The Measure of Emotional Episodes with Music (MEEM) instrument is intended to capture a person's functional use of music for the purpose of describing their emotional regulation. In total, there are twelve scales which represent separate sub-constructs. These scales are grouped into modules, with two or three scales representing different aspects of the module. There are 35 items across all five modules. Each module represents a broader theorised construct reflective of the functional use or regulatory goal a person has with music (Eerola et al., 2024).

Administration: MEEM is a state measure and is intended to differentiate between five emotional episodes to music within situational contexts. MEEM is not meant to diagnose participant traits (general tendencies of using music) but provide a contextualised description of participant's emotional episode contextualised within the situation assessed. As such, it should be administered to participants who are experiencing a scenario involving music or immediately following a musical experience. The response format should be a five point Likert type scale from Strongly disagree (1) to Strongly agree (5) with no labels placed in between for the other numbers. All of the scales for a certain module should be used together but modules can be used independently depending on research questions and study design. All items should be presented in a random order to participants, regardless of how many modules are used. Ideally, all 35 items would be presented on one page. Modifying the wording of items is not advised, the only exception would be changing spelling to reflect different English conventions. It is not advised to add or remove items unless strong empirical evidence supports the inclusion or removal of items from their target construct (Kirts et al., 2026). Additionally, MEEM can be administered in conjunction with a measure of core affect (i.e., valence and arousal) and/or a classification of the emotion experienced to further characterise the state experienced.

Scoring: Items are scored based on the keyed direction of the statements. More agreement to an item should be interpreted as supporting the statement; indicative of how a participant used the music at the moment of response. Less agreement to an item is indicative of the participant not supporting the statement but it should not be concluded they had an opposite experience against the item's meaning. No items should be reverse scored. To score responses to MEEM, take the mean of scores of all items within a module to obtain an indication of strength for that episode. It is also possible to score the individual scales within each module by calculating the mean score of each scale to obtain the strength of the individual sub-constructs. The present version of the instrument does not yet have standardised norms but comparisons within or between participants can determine percentile rankings amongst your sample of responses to MEEM.

References

Eerola, T., Kirts, C., & Saarikallio, S. (2024). Episode model: The functional approach to emotional experiences of music. *Psychology of Music*, 03057356241279763. https://doi.org/10.1177/03057356241279763

Kirts, C., Saarikallio, S., Anderson, C. J., Bannister, S., Céspedes-Guevara, J., Heng, G. J., Henry, N., Jakubowski, K., Koehler, F., Krause, A. E., Lennie, T. M., Martínez, I. C., O'Neill, K., Warrenburg, L., & Eerola, T. (2026). Measuring Emotional Experiences with Music: Content Validity Assessment for Episode Model Constructs. *Music & Science*, *9*, 20592043251413550. https://doi.org/10.1177/20592043251413550

Measure of Emotional Episodes with Music (MEEM)

Enjoyment-Distraction-Relaxation Module

Enjoyment scale

(E1)	Listening to music was a good way of entertaining myself	Strongly disagree 1 / 2 / 3 / 4 / 5 Strongly agree
(E2)	I was having a good time listening to the music	Strongly disagree 1 / 2 / 3 / 4 / 5 Strongly agree
(E3)	I used the music to have a good time	Strongly disagree 1 / 2 / 3 / 4 / 5 Strongly agree

Distraction scale

(D1)	The music helped me break away from the situation	Strongly disagree 1 / 2 / 3 / 4 / 5 Strongly agree
(D2)	Music acted as a mental shield against my distressing thoughts	Strongly disagree 1 / 2 / 3 / 4 / 5 Strongly agree
(D3)	I wanted the music to help me forget what I was doing	Strongly disagree 1 / 2 / 3 / 4 / 5 Strongly agree

Relaxation scale

(R1)	The music made me feel calm or relaxed	Strongly disagree 1 / 2 / 3 / 4 / 5 Strongly agree
(R2)	I was using the music to relax	Strongly disagree 1 / 2 / 3 / 4 / 5 Strongly agree
(R3)	Music provided a relaxing mood	Strongly disagree 1 / 2 / 3 / 4 / 5 Strongly agree

Focus-Motivation Module

Motivation Scale

(M1)	The music was motivating me to do something	Strongly disagree 1 / 2 / 3 / 4 / 5 Strongly agree
(M2)	The music pumped me up	Strongly disagree 1 / 2 / 3 / 4 / 5 Strongly agree
(M3)	I used the music to motivate myself	Strongly disagree 1 / 2 / 3 / 4 / 5 Strongly agree

Focus scale

(F1)	The music helped me concentrate	Strongly disagree 1 / 2 / 3 / 4 / 5 Strongly agree
(F2)	Music helped me focus on what I was doing	Strongly disagree 1 / 2 / 3 / 4 / 5 Strongly agree
(F3)	I was more focused while listening to the music	Strongly disagree 1 / 2 / 3 / 4 / 5 Strongly agree

Connection-Belonging Module

Group bonding scale

(G1)	I felt connected to the people I was sharing this musical experience with	Strongly disagree 1 / 2 / 3 / 4 / 5 Strongly agree
(G2)	I felt a bond with the other people listening to the music	Strongly disagree 1 / 2 / 3 / 4 / 5 Strongly agree
(G3)	The music made me feel more connected to a group	Strongly disagree 1 / 2 / 3 / 4 / 5 Strongly agree

Reducing Loneliness scale

(L1)	Listening to music made me feel less lonely	Strongly disagree 1 / 2 / 3 / 4 / 5 Strongly agree
(L2)	The music helped me feel less lonely	Strongly disagree 1 / 2 / 3 / 4 / 5 Strongly agree
(L3)	I used the music to keep me company	Strongly disagree 1 / 2 / 3 / 4 / 5 Strongly agree

Personal-Emotional-Processing Module

Comforting scale

(C1)	I found solace in the music	Strongly disagree 1 / 2 / 3 / 4 / 5 Strongly agree
(C2)	Listening to music comforted me	Strongly disagree 1 / 2 / 3 / 4 / 5 Strongly agree
(C3)	The music was comforting to me	Strongly disagree 1 / 2 / 3 / 4 / 5 Strongly agree

Expression scale

(X1)	The music helped me express myself	Strongly disagree 1 / 2 / 3 / 4 / 5 Strongly agree
(X2)	Listening to music helped me express how I was feeling	Strongly disagree 1 / 2 / 3 / 4 / 5 Strongly agree
(X3)	I vented my emotions by listening to music that expressed my feelings	Strongly disagree 1 / 2 / 3 / 4 / 5 Strongly agree

Aesthetics-Interest-Awe Module

Spirituality scale

(S1)	The music provided me with a spiritual experience	Strongly disagree 1 / 2 / 3 / 4 / 5 Strongly agree
(S2)	The music related to my spirituality	Strongly disagree 1 / 2 / 3 / 4 / 5 Strongly agree
(S3)	I wanted the music to take me somewhere spiritually	Strongly disagree 1 / 2 / 3 / 4 / 5 Strongly agree

Curiosity scale

(I1)	Listening to the music stimulated my curiosity	Strongly disagree 1 / 2 / 3 / 4 / 5 Strongly agree
(I2)	The music sparked my curiosity	Strongly disagree 1 / 2 / 3 / 4 / 5 Strongly agree

Beauty scale

(B1)	I appreciated the beauty of the music	Strongly disagree 1 / 2 / 3 / 4 / 5 Strongly agree
(B2)	The experience was enhanced by the beauty of the music	Strongly disagree 1 / 2 / 3 / 4 / 5 Strongly agree
(B3)	I had an unforgettable experience alongside the music	Strongly disagree 1 / 2 / 3 / 4 / 5 Strongly agree
